# Factors contributing to the frequent interspecies transmission of G4P[6] Rotavirus alphagastroenteritidis strains from pigs to humans in Vietnam: molecular epidemiological insights

**DOI:** 10.1099/mgen.0.001685

**Published:** 2026-04-24

**Authors:** Miho Kaneko, Thi Nguyen Hoa-Tran, Toyoko Nakagomi, Hung Manh Vu, Taichiro Takemura, Futoshi Hasebe, Anh Thi Hai Dao, Pham Hong Quynh Anh, Anh The Nguyen, Varja Grabovac, Anh Duc Dang, Noriyuki Nishida, Osamu Nakagomi

**Affiliations:** 1 Department of Cellular and Molecular Biology, Graduate School of Biomedical Sciences, Nagasaki University, Nagasaki 852-8523, Japan; 2 Department of Hygiene and Molecular Epidemiology, Graduate School of Biomedical Sciences, Nagasaki University, Nagasaki 852-8523, Japan; 3 Department of Virology, National Institute of Hygiene and Epidemiology, Hanoi 100000, Vietnam; 4 Vietnam Research Station, Institute of Tropical Medicine, Nagasaki University, Nagasaki 852-8523, Japan; 5 Vietnam Research Station, National Institute of Hygiene and Epidemiology-Nagasaki University, Hanoi 100000, Vietnam; 6 Vaccine-Preventable Diseases and Immunization Unit, Division of Programmes for Disease Control, World Health Organization Regional Office for the Western Pacific, 1000 Manila, Philippines; 7 Department of Bacteriology, National Institute of Hygiene and Epidemiology, Hanoi 100000, Vietnam

**Keywords:** G4P[6] strains, glycan-binding sites, molecular epidemiology, pig-to-human interspecies transmission, *Rotavirus alphagastroenteritidis *(RVA), Vietnam

## Abstract

In parts of Asia, including Vietnam, *Rotavirus alphagastroenteritidis* (formerly known as *Rotavirus A*; RVA) strains of porcine origin, particularly G4P[6] strains, have been detected in humans at relatively high frequencies. This study investigated the origins of G4P[6] strains identified in Vietnamese children and examined possible factors underlying their frequent detection, based on molecular epidemiological analyses. Among the 1,252 RVA-positive faecal specimens collected over a 2-year period, 28 (2.2%) contained only the G4P[6] strain. Of these, 23 (1.8%) G4P[6] strains were successfully sequenced for their whole genomes, all of which were confirmed to be entirely of porcine RVA origin, with no evidence of genomic reassortment with human RVA strains. Two distinct clusters of G4P[6] strains (comprising two and three strains, respectively) shared identical genome sequences, suggesting the possibility of limited human-to-human transmission. In contrast, the remaining 18 G4P[6] strains were genetically distinct from one another, indicating multiple, independent interspecies transmission events from pigs to humans. Phylogenetic analysis revealed that the P[6] genome segments of porcine RVA or porcine-like human RVA strains, including those identified in this study, were genetically distinct from those of typical human RVA strains and showed notable geographic variation in detection frequency. Comparative analysis of the variable regions of P[6] amino acid sequences identified characteristic residues that distinguish porcine RVA-like P[6] from human RVA-like P[6], some of which correspond to known glycan-binding sites. This study revealed a high frequency of pig-to-human spillover transmission of porcine-derived G4P[6] rotavirus strains in Vietnamese children. The findings suggest that these cases were not the result of newly emerging strains capable of sustained human-to-human transmission. Furthermore, distinct amino acid characteristics in the porcine RVA P[6] protein were identified that may play a role in facilitating the cross-species transmission of rotavirus from pigs to humans.

**Impact Statement**

*Rotavirus alphagastroenteritidis* (RVA) is a leading cause of severe gastroenteritis in young children and animals globally. Strains possessing the P[6] VP4 genotype have been detected in both humans and pigs; however, the molecular basis for their host specificity and interspecies transmission remains incompletely understood. In this study, we identified characteristic amino acid residues that distinguish human RVA-like P[6] sequences from porcine RVA-like P[6] sequences and showed that the prevalence of these different P[6] strains in humans varies geographically. Notably, some of these characteristic residues correspond to known glycan-binding sites, suggesting that the P[6] protein may play a central role in interspecies transmission and host adaptation through specific interactions with host glycans. In Vietnam, interspecies transmission of porcine-derived G4P[6] strains from pigs to humans occurs with relatively high frequency; however, strains capable of efficient human-to-human transmission have yet to emerge. Our findings suggest that the acquisition of human RVA genome segments, particularly the VP4 segment, may be necessary for such adaptation. Collectively, these findings advance our understanding of the adaptation processes of animal-derived RVA strains to human hosts and offer valuable insights for the development of preventive strategies, including rational vaccine design.

## Data Summary

The nucleotide sequences determined in this study have been deposited in the GenBank/EMBL/DDBJ databases under accession numbers LC875846–LC876283. Individual accession numbers are listed in Table S1.

## Introduction

*Rotavirus alphagastroenteritidis* (formerly known as *Rotavirus A*; RVA), a member of the *Rotavirus* genus in the *Sedoreoviridae* family, is a major cause of severe diarrhoea in infants and young animals, including humans and pigs, worldwide [1,2]. The RVA virion has a triple-layered capsid enclosing a genome composed of 11 segments of dsRNA that encode 6 structural proteins (VP1-VP4, VP6 and VP7) and six non-structural proteins (NSP1–NSP6) [3].

To classify RVA strains, a binary system is employed based on the genotypes of two outer capsid proteins: VP7 (G types) and VP4 (P types). The most prevalent human RVA strains possess G1, G2, G3, G4, G9 and G12 in combination with P[4], P[6] or P[8], with six genotype combinations being most commonly observed worldwide: G1P[8], G2P[4], G3P[8], G4P[8], G9P[8] and G12P[8] [4,5]. In addition to the conventional binary classification system, a whole-genome-based classification system developed by Matthijnssens *et al*. [6], where Gx-Px-Ix-Rx-Cx-Mx-Ax-Nx-Tx-Ex-Hx designates the genotypes of VP7-VP4-VP6-VP1-VP2-VP3-NSP1-NSP2-NSP3-NSP4-NSP5/6 genes, has become widely used in recent years. Extensive sequencing studies have identified three major genotype constellations among typical human RVA strains: the Wa-like constellation (G1/G3/G4/G9/G12-P[8]-I1-R1-C1-M1-A1-N1-T1-E1-H1), the DS-1-like constellation (G2-P[4]-I2-R2-C2-M2-A2-N2-T2-E2-H2) and the AU-1-like constellation (G3-P[9]-I3-R3-C3-M3-A3-N3-T3-E3-H3) [6,7].

RVA strains with G and/or P genotypes that differ from those of typical human RVA are also occasionally detected in humans. Such atypical G/P genotypes are thought to originate from animal RVA strains that have been introduced into human populations through interspecies transmission. RVA infections generally exhibit species specificity [8], and several biological barriers must be overcome for a virus of heterologous origin to establish sustained transmission in a new host species. A key initial barrier is the ability of the VP8* domain of the VP4 protein to bind to receptors on the host cell surface [9,10], which is considered a prerequisite for interspecies transmission. In addition, the efficiency of viral replication following cell entry represents another critical barrier, and this process is influenced by multiple viral proteins, including but not limited to the VP4 protein [11,14]. In cases where replication efficiency is suboptimal, the infection may follow a self-limiting course and could eventually be eliminated from the new host population [8].

Among the various VP4 genotypes implicated in animal-to-human interspecies transmission, the P[6] genotype is particularly noteworthy, as it is commonly found in both human RVA and porcine RVA strains. Globally, P[6] represents the third most common VP4 genotype among circulating human RVA strains. It was first recovered from neonates without diarrhoea, leading to the early suggestion that P[6] might be primarily associated with asymptomatic infections [15,17]. However, subsequent molecular epidemiological studies revealed that P[6] strains can also cause symptomatic infections in young children and are highly prevalent in certain geographic regions, particularly in Africa and South Asia [18]. Even since the 2000s, P[6] strains have remained predominant in these regions, with ~25% of human RVA strains circulating in Africa carrying the P[6] genotype [19]. In India, the proportion of P[6] strains has been reported at ~20% [20], while in Nepal, P[6] strains have accounted for 34% of cases [21] and up to 45% according to another report [22].

Porcine RVA strains share some genotypes with human RVA strains and typically exhibit the genotype constellation G3/4/5/9/11 P[6]/[7]/[13]/[19]/[23]-I1/I5-R1-C1-M1-A1/A8-N1-T1/7-E1-H1 [23,29]. Among these, G5P[7] strains are the most prevalent porcine RVA strains globally; however, there is considerable geographic variation in the distribution of circulating genotypes [1]. In several Asian countries, G3, G4 and G9 strains, which share VP7 genotypes with common human RVA strains, have each been detected more frequently than G5 strains in swine populations [28,30, 31]. Regarding VP4 genotypes, P[7] is the most commonly detected in porcine RVA strains in Asia, followed by P[6] (9.9%) and P[23] (8.7%) [1].

Numerous cases of suspected pig-to-human transmission of RVA strains have been reported worldwide [32,42]. Among these, a substantial number of strains carried the P[6] genotype, particularly in combination with G4 [32,34,37, 39]. These porcine-derived G4P[6] strains have been reported primarily in low- and middle-income countries in Asia (China, India, the Philippines, Sri Lanka, Thailand and Vietnam), as well as in Africa (the Democratic Republic of the Congo and Kenya), South America (Argentina and Paraguay) and Europe (Croatia, Hungary and Italy). With the exception of one case in China [47], all porcine-derived G4P[6] strains identified in humans and characterized through whole-genome sequencing were considered to represent independent interspecies transmission events, resulting in dead-end infections without onward transmission in the human population.

During rotavirus surveillance conducted in Vietnam with support from the World Health Organization (WHO), we confirmed that G4P[6] strains were consistently detected in children over a 2-year period from July 2015 to June 2017. Given that porcine-derived G4P[6] strains have often been detected in humans across various Asian countries and that all five G4P[6] strains previously identified in Vietnamese children were confirmed to be of porcine origin in our earlier study [42], we inferred that the G4P[6] strains identified in the present study would likely be of porcine origin. Assuming that this prediction is correct, the detection rate of porcine-derived G4P[6] strains would be ≥2%, a percentage higher than what has generally been perceived. This observation prompted us to ask why interspecies transmission of G4P[6] strains from pigs to humans occurred relatively frequently in Vietnam and came to two possible working hypotheses: (i) the emergence of a novel G4P[6] strain enabling efficient human-to-human transmission and/or (ii) the existence of some biological or epidemiological conditions in Vietnam that facilitate pig-to-human spillover events.

In this study, to evaluate the plausibility of these two working hypotheses, we performed a comprehensive molecular epidemiological characterization of the detected P[6] strains. In parallel, we compared the amino acid sequences of the variable regions of the VP8* domain of the VP4 protein between human-derived and porcine-derived P[6] strains available in the GenBank/EMBL/DDBJ databases and those obtained in this study, given the pivotal role of the VP4 protein in host range determination. Through these approaches, we aimed to identify potential factors that could explain the relatively frequent interspecies transmission of G4P[6] strains from pigs to humans in Vietnam.

## Methods

### Study samples

As part of the WHO-funded rotavirus surveillance programme, a total of 3,132 faecal specimens were collected from children under 5 years of age who visited or were admitted to sentinel hospitals in northern and central Vietnam for acute gastroenteritis between July 2015 and June 2017. All samples were tested for RVA antigen using an ELISA with the ProSpecT Rotavirus kit (Thermo Fisher Scientific, Waltham, MA, USA), and 1,252 (40%) were found to be RVA-positive. Demographic data of the enrolled patients were collected, along with information on signs and symptoms at the time of presentation to the hospitals.

This surveillance programme has also generated reports of other unusual genotype constellations [52,54]. Although the surveillance periods partially overlapped, none of the strains analysed in this study were included in those previous studies.

### G and P genotyping

G and P genotyping had already been completed for ~70% of the 1,252 RVA-positive specimens prior to the start of this study. The remaining specimens were genotyped to identify all strains possessing G4 and/or P[6] genotypes for inclusion as study samples. Genomic RNA was extracted from 20% suspensions of RVA-positive faecal specimens in PBS using the QIAamp Viral RNA Mini Kit (Qiagen, Venlo, The Netherlands). The extracted RNA was then used for reverse transcription PCR (RT-PCR) with the QIAamp OneStep RT-PCR Kit (Qiagen) and the first primer set. The first PCR products were added at a 1:100 dilution to the second PCR reaction mixture. The second PCR was performed using either AmpliTaq DNA polymerase (Applied Biosystems, Waltham, MA, USA) or GoTaq Green Master Mix (Promega, Madison, WI, USA) along with typing primers. Two different methods were applied for G-typing and P-typing, respectively, as summarized in Table S2 (available in the online Supplementary Material), based on the revision of the genotyping protocol in the National Institute of Hygiene and Epidemiology of Vietnam in 2017. For G-typing, genotypes G1, G2, G3, G4, G8 and G9 could be identified using either method, whereas G12 could only be typed using G-typing Method B (Table S2). Similarly, for P-typing, genotypes P[4], P[6], P[8], P[9] and P[10] could be identified using either method, while P[11] could only be typed using P-typing Method A (Table S2). If at least one of the VP7 and VP4 genotypes remained untypeable, we revisited the first RT-PCR products of the corresponding genes and determined their nucleotide sequences by Sanger sequencing, followed by genotyping using the RVA Genotyping Tool in the Virus Pathogen Resource (ViPR, https://www.bv-brc.org/). Samples in which G-P typing results revealed mixed infection with multiple RVA strains, as well as samples that remained untyped for either VP7 or VP4, were excluded from subsequent whole-genome analyses.

### Whole-genome sequencing

Before cDNA library construction, contaminating DNAs were removed from the extracted RNAs by DNase treatment using the TURBO DNA-free Kit (Thermo Fisher Scientific). cDNA libraries consisting of 300 bp fragments ligated with barcoded adapters were prepared using the NEBNext Ultra RNA library Prep Kit for Illumina (New England Biolabs, Ipswich, MA, USA) and the NEBNext Multiplex Oligos for Illumina (New England Biolabs) according to the manufacturer’s instructions and followed by purification with Agencourt AMPure XP magnetic beads (Beckman Coulter, Brea, CA, USA). The concentrations and quality of the purified libraries were assessed using the Qubit dsDNA HS Assay kit (Thermo Fisher Scientific) on a Qubit 2.0 fluorometer (Thermo Fisher Scientific) and by agarose gel electrophoresis. Nucleotide sequencing was performed on an Illumina MiSeq sequencer (Illumina, San Diego, CA, USA) using the MiSeq Reagent Kit v2 (300 cycles) (Illumina) to generate 151 bp paired-end reads. Contigs were generated by *de novo* assembly using CLC Genomics Workbench Software v7.0.3 (CLC Bio, Aarhus, Denmark).

### Sequence and phylogenetic analyses

Multiple alignments of nucleotide sequences were performed using the online version of the Multiple Alignment using the Fast Fourier Transform (MAFFT) program (v7) [55]. For sequence comparisons and phylogenetic analyses, sequences were retrieved from the GenBank/EMBL/DDBJ databases through blast searches. Phylogenetic trees for all 11 genome segments were constructed using the maximum likelihood (ML) method with the best-fit substitution models, selected based on the lowest Bayesian information criterion (BIC) scores: T92+G+I (G3/G5/G9 VP7, P[6] VP4, VP6, NSP2), T92+G (NSP3, NSP5), GTR+G (G4 VP7, VP1), GTR+G+I (VP2, VP3, NSP1) and HKY+G+I (NSP4). Bootstrap analysis was performed with 1,000 replicates, and bootstrap values ≥70% are indicated at each node. Scale bars at the bottom of the trees represent genetic distance (nucleotide substitutions per site).

For the G4 VP7 phylogenetic analysis, all available G4 sequences of RVA strains detected in humans or pigs/wild boars with lengths ≥920 bp were retrieved from the GenBank/EMBL/DDBJ databases. A preliminary Neighbour-Joining (NJ) tree was constructed using this dataset. Human-derived RVA strains that fell outside the cluster of typical Wa-like human RVA strains were excluded from the final ML tree, unless their origins had been previously characterized. For the G3/G5/G9 VP7 phylogenetic analysis, the G3 sequence set was expanded by incorporating relevant human and porcine RVA sequences into the representative strain set established by Kaneko *et al*. [42]. Representative G9 sequences were selected with reference to the datasets provided in the previous studies [53,56,58]. For G5, additional published sequences were incorporated into the set of strains carrying the G5 genotype, which was used in the phylogenetic analysis of genotype 1 genes as described below.

To construct the phylogenetic tree for P[6] VP4 sequences, all P[6] sequences with ≥2,000 bp in length that fully covered the 202–678 of the nucleotide sequences (corresponding to the variable region of the VP8* protein, amino acids 65–223) of the VP4 genome segment were retrieved from the GenBank/EMBL/DDBJ databases (491 sequences). A preliminary NJ tree was constructed, in which a single cluster consisting exclusively of human RVA strains was observed. Of the human-derived RVA strains falling outside this cluster, ten strains were excluded from the final ML tree because their origins had never been discussed in published literature. As a result, 481 P[6] sequences were included in the ML tree.

For the genotype 1 genes of the VP1–VP3 and NSP2–NSP5 genome segments, ML trees were constructed using nucleotide sequences from four groups of strains: (i) typical modern human RVA strains, (ii) archival human RVA strains, (iii) porcine/wild boar RVA strains, and (iv) porcine-like human RVA strains. As representatives of typical modern human RVA strains, we included 33 strains selected by Silva *et al*. [59] along with three Vietnamese G9P[8] strains, which were part of the same sample collection in this study and had been previously characterized [53]. As representatives of archival human RVA strains, six additional strains were added to the five selected by Silva *et al*. [59], all isolated before 1994. Porcine/wild boar RVA strains included those from pigs or wild boars whose genome sequences had been nearly fully determined and published. Porcine-like human RVA strains were defined as human-derived RVA strains previously shown to possess the majority of their genome segments of porcine origin. As described in a previous study [59], the genotype 1 genes of most typical modern human RVA strains form distinct clusters, separate from those of porcine/wild boar RVA strains and porcine-like human RVA strains. Archival human RVA strains clustered with typical modern human RVA strains in some genome segments, while in others, they formed clusters exclusively with other archival human RVA strains or with porcine/wild boar and porcine-like human RVA strains. A genome segment of a strain in question was considered to be of porcine RVA origin if: (i) it did not belong to clusters consisting exclusively or predominantly of typical modern human RVA strains and (ii) it was more closely related to porcine/wild boar RVA strains or porcine-like human RVA strains than to typical modern human RVA strains, based on both phylogenetic relationships and nucleotide sequence identity.

For the I5, A8 and T7 genotypes, only sequences from strains possessing these genotypes were used to construct each phylogenetic tree. These strains were selected from the set used for constructing the genotype 1 phylogenetic trees.

### Identification of amino acid residues distinguishing porcine RVA-like P[6] from human RVA-like P[6]

**Fig. 1.**
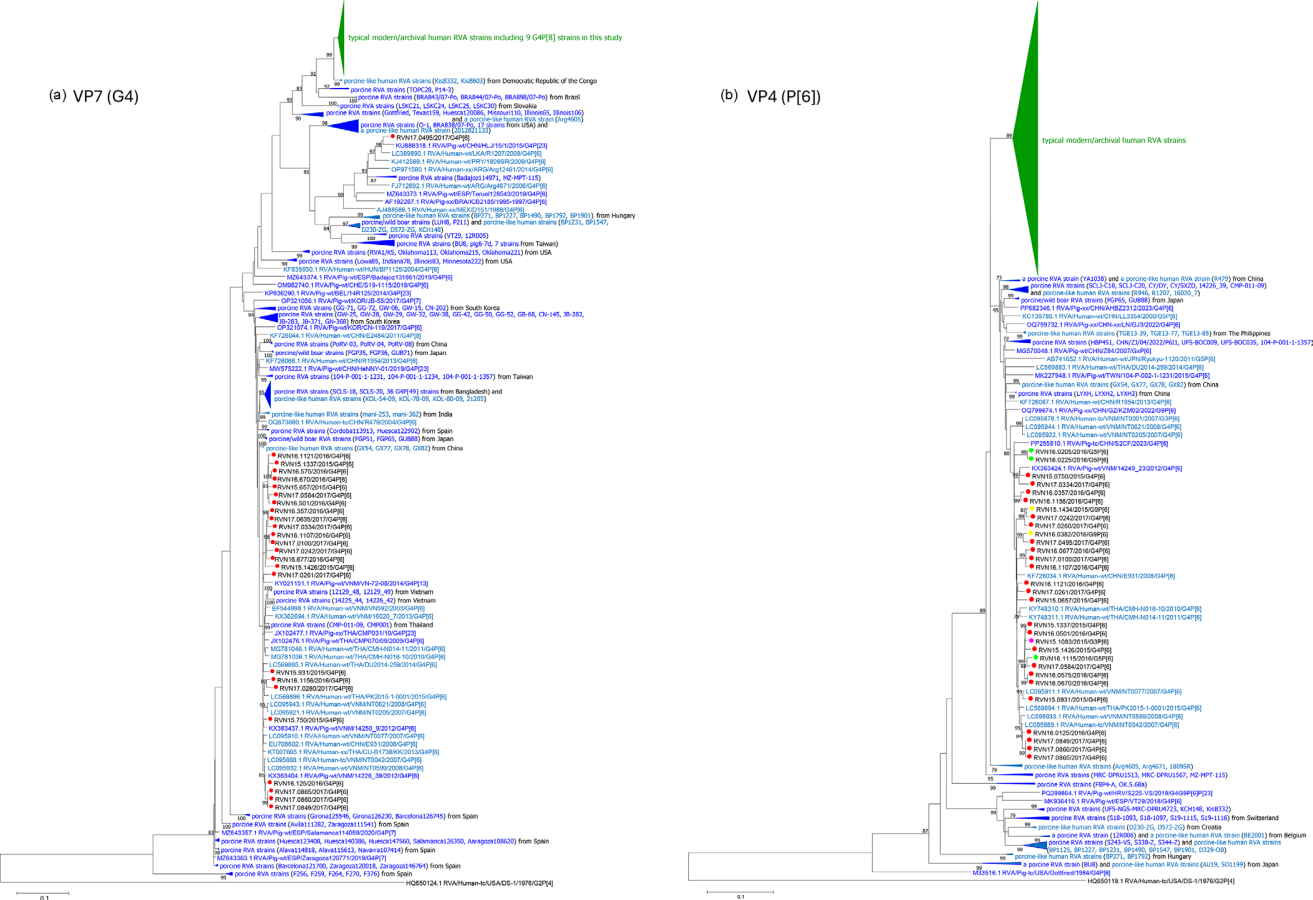
ML phylogenetic trees of the G4 VP7 (**a**) and P[6] VP4 (**b**) genes from Vietnamese G4 and/or P[6] RVA strains and reference RVA strains. Phylogenetic trees were constructed using the ML method with 1,000 bootstrap replicates in mega7. Bootstrap values ≥70% are shown at the corresponding nodes. Scale bars indicate genetic distance, expressed as the number of nucleotide substitutions per site. Strain names of Vietnamese isolates sequenced in this study are shown in black, with coloured filled circles indicating their G and P genotypes. Reference RVA strains are colour-coded based on the host species in which they were detected and their presumed origin: human RVA strains from humans (green), porcine RVA strains from pigs (blue) and porcine-like RVA strains detected in humans (light blue).

For the 481 P[6] nucleotide sequences included in the ML phylogenetic tree (Fig. 1b), the 202–678 nt region (corresponding to the variable region of the VP8* protein, amino acids 65–223) was translated into amino acid sequences. Based on these partial sequences, a phylogenetic tree was reconstructed using the ML method with the best-fit substitution model determined by the lowest BIC score: JTT+G (Fig. S2). Bootstrap analysis was performed with 1,000 replicates, and bootstrap values ≥60% are indicated at each node. Based on their positions in this ML tree, the P[6] sequences were classified into two groups: human RVA-like P[6] and porcine RVA-like P[6]. Subsequently, the most frequent amino acid residue at each position within each group was identified to generate a consensus sequence for that group. These consensus sequences were then compared between the two groups. Differences in amino acid residues between the consensus sequences were recorded, and the degree of conservation within each group as well as the similarity in amino acid properties between the two groups was evaluated.

## Results

Among the 1,252 RVA-positive specimens collected from children with diarrhoea in northern and central Vietnam between July 2015 and June 2017, 44 (3.5%) contained RVA strains with the G4 genotype (Table 1), and 45 (3.6%) contained RVA strains with the P[6] genotype (Table 2). A single G and P genotype combination was identified in 43 of these specimens: 9 (0.7% of all RVA-positive samples) were G4P[8] strains (Table 1), and 34 (2.7% of all RVA-positive samples) were P[6] strains in combination with either G1, G3, G4 or G9 (Table 2). Notably, among the 34 P[6] strains, 28 (82% of P[6] strains; corresponding to 2.2% of all RVA-positive samples) carried G4 as the VP7 genotype. These 43 specimens with a single G4 and/or P[6] genotype combination were subjected to whole-genome sequencing under the assumption that each infection was caused by a single strain.

**Table 1.** Number of samples containing RVA strains with the G4 genotype

	P[6]	P[6]+P[8]	P[8]	P[others]	P[untypeable]	Total
G4	**28** (2.2%)*	0	**9** (0.7%)	0	0	**37** (3.0%)
G1+G2+G4	0	0	1 (0.1%)	0	0	1 (0.1%)
G2+G4	0	0	1 (0.1%)	0	0	1 (0.1%)
G2+G4+G8	0	1 (0.1%)	1 (0.1%)	0	0	2 (0.2%)
G4+G8	1 (0.1%)	0	0	0	0	1 (0.1%)
G4+G9	2 (0.2%)	0	0	0	0	2 (0.2%)
Total	31 (2.5%)	1 (0.1%)	12 (1.0%)	0	0	44 (3.5%)

Strains shown in bold are those with a single G and P genotype that were subjected to whole-genome sequencing. Numbers in parentheses indicate the percentage of each genotype relative to the 1,252 RVA-positive samples. The 28 G4P[6] strains marked with an asterisk (*) overlap with those listed in Table 2.

**Table 2.** Number of samples containing RVA strains with the P[6] genotype

	P [6]	P [6]+P [8]	Total
G3	**1** (0.1%)	0	1 (0.1%)
G4	**28** (2.2%)*	0	28 (2.2%)
G5	**3** (0.2%)	0	3 (0.2%)
G9	**2** (0.2%)	2 (0.2%)	4 (0.3%)
G2+G4+G8	0	1 (0.1%)	1 (0.1%)
G2+G9	0	1 (0.1%)	1 (0.1%)
G3+G8	2 (0.2%)	0	2 (0.2%)
G4+G8	1 (0.1%)	0	1 (0.1%)
G4+G9	2 (0.2%)	0	2 (0.2%)
G others	0	0	0
G untypeable	1 (0.1%)	1 (0.1%)	2 (0.2%)
Total	40 (3.2%)	5 (0.4%)	45 (3.6%)

Strains shown in bold are those with a single G and P genotype that were subjected to whole-genome sequencing. Numbers in parentheses indicate the percentage of each genotype relative to the 1,252 RVA-positive samples. The 28 G4P[6] strains marked with an asterisk (*) overlap with those listed in Table 1.

Between two and seven cases with G4P[6] strains were detected each quarter, with relatively high detection rates observed during seasons with a low overall prevalence of rotavirus diarrhoea (Fig. S3).

### Genotype constellation of the sequenced strains

Of the 43 strains subjected to the next-generation sequencing, the genotype constellations of 41 strains were determined. These included 26 G4P[6], 1 G3P[6], 3 G5P[6], 2 G9P[6] and 9 G4P[8] strains (Table 3). Among the G4P[6] strains, the most common genotype constellation was G4-P[6]-I1-R1-C1-M1-A8-N1-T1-E1-H1, observed in 14 strains. The second most common constellation, identified in 11 strains, was G4-P[6]-I1-R1-C1-M1-A8-N1-T7-E1-H1. One strain exhibited the constellation G4-P[6]-I5-R1-C1-M1-A8-N1-T1-E1-H1.

**Table 3.** Genotype constellations of the G4 and/or P[6] strains characterized in this study

Strain	VP7	VP4	VP6	VP1	VP2	VP3	NSP1	NSP2	NSP3	NSP4	NSP5
RVN15.657	G4	P[6]	I1	R1	C1	M1	A8	N1	T1	E1	H1
RVN15.931	G4	P[6]	I1	R1	C1	M1	A8	N1	T1	E1	H1
RVN15.1337	G4	P[6]	I1	R1	C1	M1	A8	N1	T1	E1	H1
RVN16.125	G4	P[6]	I1	R1	C1	M1	A8	N1	T1	E1	H1
RVN16.677	G4	P[6]	I1	R1	C1	M1	A8	N1	T1	E1	H1
RVN16.1107	G4	P[6]	I1	R1	C1	M1	A8	N1	T1	E1	H1
RVN16.1396	G4	P[6]	I1	R1	C1	M1	A8	N1	T1	E1	H1
RVN17.0100	G4	P[6]	I1	R1	C1	M1	A8	N1	T1	E1	H1
RVN17.0260	G4	P[6]	I1	R1	C1	M1	A8	N1	T1	E1	H1
RVN17.0261	G4	P[6]	I1	R1	C1	M1	A8	N1	T1	E1	H1
RVN17.0334	G4	P[6]	I1	R1	C1	M1	A8	N1	T1	E1	H1
RVN17.0495	G4	P[6]	I1	R1	C1	M1	A8	N1	T1	E1	H1
RVN17.0584	G4	P[6]	I1	R1	C1	M1	A8	N1	T1	E1	H1
RVN17.0635	G4	P[6]	I1	R1	C1	M1	A8	N1	T1	E1	H1
RVN15.750	G4	P[6]	I1	R1	C1	M1	A8	N1	T7	E1	H1
RVN15.1426	G4	P[6]	I1	R1	C1	M1	A8	N1	T7	E1	H1
RVN16.357	G4	P[6]	I1	R1	C1	M1	A8	N1	T7	E1	H1
RVN16.501	G4	P[6]	I1	R1	C1	M1	A8	N1	T7	E1	H1
RVN16.575	G4	P[6]	I1	R1	C1	M1	A8	N1	T7	E1	H1
RVN16.670	G4	P[6]	I1	R1	C1	M1	A8	N1	T7	E1	H1
RVN16.1121	G4	P[6]	I1	R1	C1	M1	A8	N1	T7	E1	H1
RVN16.1156	G4	P[6]	I1	R1	C1	M1	A8	N1	T7	E1	H1
RVN17.0849	G4	P[6]	I1	R1	C1	M1	A8	N1	T7	E1	H1
RVN17.0860	G4	P[6]	I1	R1	C1	M1	A8	N1	T7	E1	H1
RVN17.0865	G4	P[6]	I1	R1	C1	M1	A8	N1	T7	E1	H1
RVN17.0242	G4	P[6]	I5	R1	C1	M1	A8	N1	T1	E1	H1
RVN15.1083	G3	P[6]	I1	R1	C1	M1	A1	N1	T1	E1	H1
RVN16.205	G5	P[6]	I5	R1	C1	M1	A8	N1	T7	E1	H1
RVN16.225	G5	P[6]	I5	R1	C1	M1	A8	N1	T7	E1	H1
RVN16.1115	G5	P[6]	I5	R1	C1	M1	A8	N1	T1	E1	H1
RVN15.1434	G9	P[6]	I5	R1	C1	M1	A8	N1	T7	E1	H1
RVN16.382	G9	P[6]	I5	R1	C1	M1	A8	N1	T1	E1	H1
RVN17.0155	G4	P[8]	I1	R1	C1	M1	A1	N1	T1	E1	H1
RVN17.0347	G4	P[8]	I1	R1	C1	M1	A1	N1	T1	E1	H1
RVN17.0681	G4	P[8]	I1	R1	C1	M1	A1	N1	T1	E1	H1
RVN17.0687	G4	P[8]	I1	R1	C1	M1	A1	N1	T1	E1	H1
RVN17.0693	G4	P[8]	I1	R1	C1	M1	A1	N1	T1	E1	H1
RVN17.0696	G4	P[8]	I1	R1	C1	M1	A1	N1	T1	E1	H1
RVN17.0801	G4	P[8]	I1	R1	C1	M1	A1	N1	T1	E1	H1
RVN17.0804	G4	P[8]	I1	R1	C1	M1	A1	N1	T1	E1	H1
RVN17.0813	G4	P[8]	I1	R1	C1	M1	A1	N1	T1	E1	H1

Genotypes characteristic of porcine RVA strains are highlighted in cyan.

Sequences that were genotyped but not included in the phylogenetic analysis are highlighted in grey.

Two G4P[6] strains could not be sequenced for any genome segment. In addition, three G4P[6] strains contained genomic segments that could not be assembled into single contigs due to undetermined regions. These included the VP4, VP1 and VP2 genome segments of strain RVN16.1396; the NSP5 genome segment of strain RVN15.931; and the VP4 genome segment of strain RVN17.0635. However, for each of these segments, partial ORF sequences exceeding 500 bp were successfully determined, enabling genotyping via the ViPR tool.

Other P[6] strains also had the genotype constellations comprising I1/I5-R1-C1-M1-A1/A8-N1-T1/T7-E1-H1 for the internal capsid and nonstructural genes. While all other P[6] strains carried at least one genotype typically associated with porcine RVA strains (i.e. I5, A8 and T7), the G3P[6] strain had only genotype 1 genes in these genome segments, matching the backbone genotype constellation of typical modern Wa-like human RVA strains (i.e. I1-R1-C1-M1-A1-N1-T1-E1-H1).

All nine G4P[8] strains shared the genotype constellation G4-P[8]-I1-R1-C1-M1-A1-N1-T1-E1-H1.

Due to the incomplete sequencing, the NSP5 sequence of strain RVN15.931, the VP4 sequence of strain RVN17.0635 and the entire genome sequences of strain RVN16.1396 were excluded from subsequent phylogenetic analysis (Table 3).

### Phylogenetic analysis of the VP7 genes

In the G4 phylogenetic tree (Fig. 1a), a distinct cluster was formed, comprising exclusively typical modern and archival human RVA strains, including nine G4P[8] strains identified in this study. In contrast, porcine RVA strains and porcine-like human RVA strains formed several genetically divergent clusters. All G4P[6] strains identified in this study, with the exception of strain RVN17.0495, belonged to one of these porcine RVA-related clusters. Although strain RVN17.0495 was genetically distant from the other G4P[6] strains identified here, it also grouped within a cluster composed of porcine RVA strains and porcine RVA-like human strains.

In the phylogenetic tree constructed using a combined set of G3, G5 and G9 sequences (Fig. S1A), the P[6] strains identified in this study clustered with porcine RVA and/or porcine-like human RVA strains sharing the same G genotype.

### Phylogenetic analysis of the VP4 genes

In the P[6] phylogenetic tree (Fig. 1b), a major cluster was formed with 89% bootstrap support, consisting exclusively of typical modern and archival human RVA strains. Outside this cluster, several smaller and genetically diverse clusters were observed, comprising porcine RVA strains and/or porcine-like human RVA strains. All P[6] sequences identified in this study were grouped within one of these porcine RVA-related clusters, which was supported by an 80% bootstrap value. Notably, all strains within this cluster were detected in Asian countries. This cluster also included six P[6] strains detected in Vietnamese children in our previous study [42], as well as strain 14249_23 detected in a Vietnamese pig [43], suggesting a close genetic relationship among these strains.

### Phylogenetic analysis of the internal capsid and nonstructural genes

Phylogenetic analyses of genotype 1 genes (I1 VP6, R1 VP1, C1 VP2, M1 VP3, A1 NSP1, N1 NSP2, T1 NSP3, E1 NSP4 and H1 NSP5) revealed that the nine G4P[8] strains identified in this study consistently clustered with typical modern human RVA strains, often alongside several archival human RVA strains (Fig. S1B–J). In contrast, all 29 P[6] strains identified in this study with nearly complete genome sequences consistently clustered with porcine RVA strains and/or porcine-like human RVA strains across all genome segments.

Notably, the N1 NSP2 phylogenetic tree exhibited a more complex structure than those of other genotype 1 gene trees, with typical modern human RVA strains dispersed across multiple lineages (Fig. S1G). Moreover, one cluster included both typical human RVA strains, including the nine G4P[8] strains from this study and porcine-like human RVA strains (R946, R1954 and 07N1760). Nevertheless, the P[6] strains identified in this study remained more closely related to porcine RVA and porcine-like human RVA strains.

Since I5 VP6, A8 NSP1 and T7 NSP3 are genotypes characteristic of porcine RVA strains, the phylogenetic trees for these sequences consisted exclusively of porcine RVA and porcine-like human RVA strains (Fig. S1K–M). In these trees, P[6] strains identified in this study exhibited close genetic relatedness to strains detected in Asia, regardless of their G and P genotype combinations.

### Three sets of identical P[6] strains

Nucleotide sequence comparison revealed three sets of P[6] strains with identical genomes: RVN16.205 and RVN16.225 (G5P[6]); RVN16.575 and RVN16.670 (G4P[6]); and RVN17.0849, RVN17.0860 and RVN17.0865 (G4P[6]) (Table 4). The two G5P[6] strains (RVN16.205 and RVN16.225) and the three G4P[6] strains (RVN17.0849, RVN17.0860 and RVN17.0865) shared 100% nucleotide identity across all genome segments, respectively. Another pair of G4P[6] strains, RVN16.575 and RVN16.670, also exhibited complete identity across all genome segments except for NSP5, which showed 99.7% identity.

**Table 4.** Demographic data of patients infected with identical porcine-derived P[6] RVA strains

Strain	Genotype constellation	Age (month)	Date of onset	Residence	
				District/Municipality	Province
RVN16.205*	G5-P[6]-I5-R1-C1-M1-A8-N1-T7-E1-H1	10	29 February 2016	Hoai Duc	Hanoi
RVN16.225*	G5-P[6]-I5-R1-C1-M1-A8-N1-T7-E1-H1	16	6 March 2016	Ha Dong	Hanoi
RVN16.575**	G4-P[6]-I1-R1-C1-M1-A8-N1-T7-E1-H1	4	22 June 2016	Ha Dong	Hanoi
RVN16.670**	G4-P[6]-I1-R1-C1-M1-A8-N1-T7-E1-H1	59	8 August 2016	Moc Chau	Son La
RVN17.0849***	G4-P[6]-I1-R1-C1-M1-A8-N1-T7-E1-H1	45	2 June 2017	Ninh Ich	Khanh Hoa
RVN17.0860***	G4-P[6]-I1-R1-C1-M1-A8-N1-T7-E1-H1	14	22 June 2017	Ninh Xuan	Khanh Hoa
RVN17.0865***	G4-P[6]-I1-R1-C1-M1-A8-N1-T7-E1-H1	22	29 June 2017	Ninh Than	Khanh Hoa

Strains sharing the same number of asterisks exhibit identical or nearly identical genome sequences.

No epidemiological links were identified among the patients infected with these strains. The patients with RVN16.205 and RVN16.225 resided in different districts of Hanoi Province. Similarly, those infected with RVN17.0849, RVN17.0860 and RVN17.0865 were located in separate districts of Khanh Hoa Province. The patient with RVN16.575 was from Ha Dong District in Hanoi Province, whereas the patient with RVN16.670 lived in Moc Chau District, Son La Province, ~180 km apart. The onset of symptoms in these two cases occurred 47 days apart.

### Differences in amino acid sequences in the variable region of VP8*s between human RVA-like P[6] and porcine RVA-like P[6] sequences

Since the human RVA P[6] sequences and the porcine RVA/porcine-like human RVA P[6] sequences formed distinct clusters in the phylogenetic tree (Fig. 1b), we hypothesized that specific sequence differences may exist that distinguish the two groups. Given the critical role of VP4 in viral entry and the fact that most strains within the porcine-like human RVA P[6] cluster, including the Vietnamese P[6] strains identified in this study, were detected in Asian countries, we further postulated that porcine RVA-like P[6] sequences may harbour specific features that facilitate infection in Asian populations.

Since the human RVA P[6] sequences and the porcine RVA/porcine-like human RVA P[6] sequences formed distinct clusters in the phylogenetic tree (Fig. 1b), we hypothesized that specific sequence differences may exist that distinguish the two groups. Given the critical role of VP4 in viral entry and the fact that most strains within the porcine-like human RVA P[6] cluster, including the Vietnamese P[6] strains identified in this study, were detected in Asian countries, we further postulated that porcine RVA-like P[6] sequences may harbour specific features that facilitate infection in Asian populations.

We next analysed the geographic distribution of the human-derived P[6] strains. Porcine RVA-like human P[6] strains (75 strains) were predominantly detected in several Southeast and East Asian countries, including Vietnam, as well as in certain European countries (Table 5). In contrast, the majority of P[6] strains identified in African and South Asian populations belonged to the human RVA-like P[6] group (Table 5).

**Table 5.** Classification of 440 P[6] strains detected in humans based on their variable region sequences, shown by country of detection

Region	Country	Human RVA-like P[6]		Porcine RVA-like P[6]		Total no. of strains
		No. of strains	Detection rate (%)	No. of strains	Detection rate (%)	
Southeast Asia	Vietnam	0	0.0	37	100.0	37
	The Philippines	0	0.0	3	100.0	3
	Thailand	4	50.0	4	50.0	8
	Indonesia	6	100.0	0	0.0	6
	Myanmar	1	100.0	0	0.0	1
East Asia	Japan	0	0.0	3	100.0	3
	China	12	57.1	9	42.9	21
	South Korea	7	100.0	0	0.0	7
Europe	Hungary	0	0.0	8	100.0	8
	Croatia	0	0.0	3	100.0	3
	Belgium	3	75.0	1	25.0	4
	Russia	4	100.0	0	0.0	4
	Germany	1	100.0	0	0.0	1
	Italy	1	100.0	0	0.0	1
	Sweden	1	100.0	0	0.0	1
	UK	1	100.0	0	0.0	1
Central and South America	Paraguay	0	0.0	1	100.0	1
	Argentine	2	50.0	2	50.0	4
	Brazil	4	100.0	0	0.0	4
	Venezuela	3	100.0	0	0.0	3
	Suriname	1	100.0	0	0.0	1
	Dominican Republic	1	100.0	0	0.0	1
North America	USA	14	100.0	0	0.0	14
Africa	Zambia	3	75.0	1	25.0	4
	Democratic Republic of the Congo	5	83.3	1	16.7	6
	Kenya	10	90.9	1	9.1	11
	South Africa	33	100.0	0	0.0	33
	Mozambique	20	100.0	0	0.0	20
	Ghana	23	100.0	0	0.0	23
	Malawi	20	100.0	0	0.0	20
	Mali	18	100.0	0	0.0	18
	Benin	16	100.0	0	0.0	16
	Cameroon	15	100.0	0	0.0	15
	Uganda	10	100.0	0	0.0	10
	Zimbabwe	9	100.0	0	0.0	9
	Ethiopia	6	100.0	0	0.0	6
	Togo	6	100.0	0	0.0	6
	Gambia	4	100.0	0	0.0	4
	Guinea-Bissau	4	100.0	0	0.0	4
	Senegal	3	100.0	0	0.0	3
	Botswana	1	100.0	0	0.0	1
	Central African Republic	1	100.0	0	0.0	1
	Egypt	1	100.0	0	0.0	1
	Eswatini	1	100.0	0	0.0	1
South Asia	Sri Lanka	0	0.0	1	100.0	1
	Nepal	35	100.0	0	0.0	35
	India	25	100.0	0	0.0	25
	Bangladesh	15	100.0	0	0.0	15
	Pakistan	4	100.0	0	0.0	4
Middle East	Lebanon	8	100.0	0	0.0	8
Oceania	Australia	3	100.0	0	0.0	3
Total		365	83.0	75	17.0	440

**Fig. 2.**
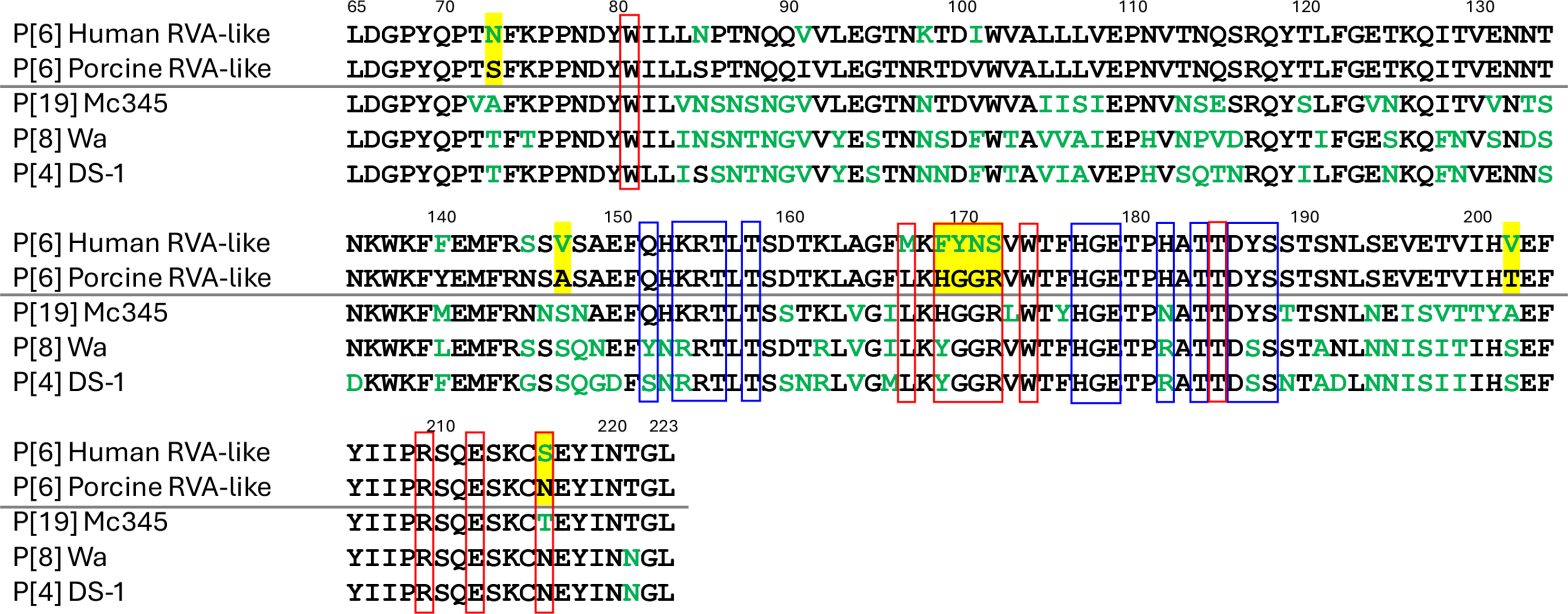
Sequence alignment of the variable region (residues 65–223) of the VP8* domains of the VP4 protein. Consensus sequences representing the most frequently observed amino acids among human RVA-like P[6] strains and porcine RVA-like P[6] strains were independently generated and aligned with representative sequences of P[8] (Wa strain), P[4] (DS-1 strain) and P[19] (Mc345 strain). Amino acid residues that differ from the porcine RVA-like P[6] consensus sequence are shown in green. The *βα* and the *ββ* glycan-binding sites of the VP8* domain [85] are outlined in red and blue, respectively. Residues showing marked divergence between the human RVA-like P[6] and porcine RVA-like P[6] consensus sequences, defined as being highly conserved within each group (≥70%) but exhibiting low or no similarity between groups, are highlighted in yellow.

To further elucidate the differences between porcine RVA-like P[6] and human RVA-like P[6], we compared the consensus sequences, defined as the most frequently observed amino acid residues at each position within each group (Fig. 2). Because P[6] is classified within the P[II] genogroup together with P[4], P[8] and P[19] based on VP8* phylogeny [60], representative strains of these genotypes were also included to provide evolutionary context. Among them, the human porcine-origin P[19] strain Mc345 was included as a reference. A total of 15 amino acid positions differed between the two P[6] groups (Table 6). Among these, eight residues were highly conserved within each group, being present in more than 70% of the sequences, but showed low or no similarity in amino acid properties between the groups (Table 6). Notably, five of these eight residues at positions 169, 170, 171, 172 and 216 have previously been proposed to function as glycan binding on the host cell surface [61]. In particular, positions 169 to 172 exhibited pronounced differences between the groups: nearly all human RVA-like P[6] sequences encoded F169, Y170, N171 and S172, whereas all porcine RVA-like P[6] sequences encoded H169, G170, G171 and R172 (Table 6).

**Table 6.** Fifteen amino acid residues in the variable region of the VP8* (amino acids 65–223) showing differential properties and frequency between human RVA-like P[6] and porcine RVA-like P[6] sequences

Amino acid position	Human RVA-like P[6] (*n*=365)		Porcine RVA-like P[6] (*n*=116)		Similarity of amino acid properties
	Most common amino acid residue	No. of sequences	Most common amino acid residue	No. of sequences	
**73**	N	303 (83.0%)	S	108 (93.1%)	Low
85	N	363 (99.5%)	S	69 (59.5%)	Low
91	V	363 (99.5%)	I	104 (89.7%)	High
98	K	255 (69.9%)	R	102 (87.9%)	High
101	I	259 (71.0%)	V	112 (96.6%)	High
140	F	321 (87.9%)	Y	79 (68.1%)	High
145	S	185 (50.7%)	N	104 (89.7%)	Low
**147**	V	329 (90.1%)	A	98 (84.5%)	Low
167*	M	350 (95.9%)	L	116 (100.0%)	High
169*	F	351 (96.2%)	H	116 (100.0%)	Low
170*	Y	364 (99.7%)	G	116 (100.0%)	No
171*	N	365 (100.0%)	G	116 (100.0%)	Low
172*	S	365 (100.0%)	R	116 (100.0%)	No
**202**	V	364 (99.7%)	T	107 (92.2%)	Low
216*	S	332 (91.0%)	N	85 (73.3%)	Low

Amino acid positions shown in bold indicate residues exhibiting significant differences between human RVA-like P[6] and porcine RVA-like P[6] sequences, based on high conservation within groups (≥70%) and low or no similarity between groups.

Amino acid positions marked with an asterisk (*) denote residues previously reported to be involved in glycan binding.

## Discussion

Our genomic analyses demonstrate that all Vietnamese P[6] RVA strains identified in this study are of porcine origin.

Based on accumulated reports from various regions, porcine RVA strains with the G4P[6] genotype combination appear to be among the most important contributors to pig-to-human interspecies transmission worldwide. Several studies to date have allowed us to calculate the proportion of porcine G4P[6] strains among the causative strains of rotavirus diarrhoea in children. The detection rates of G4P[6] strains, for which whole-genome analyses concluded that all or most genome segments were of porcine RVA origin, are as follows: China (1.1%, 1/90) [62], India (0.41%, 1/244) [63], Paraguay (0.97%, 1/103) [45], Sri Lanka (1.4%, 1/74) [41], Vietnam (1.6%, 5/322) [42] and the Philippines (1.3%, 2/151) [37]. The detection rate of 1.8% (23/1,251) obtained in this study was the highest reported to date, with a 95% lower confidence limit of 1.2%, the only one in which the lower confidence limit exceeded 1%.

Consequently, to better understand why porcine-derived G4P[6] strains have a relatively high impact on humans in Vietnam, we investigated the issue from a molecular epidemiological perspective. Given the repeated detection of G4P[6] strains within a limited temporal and geographical window, we first examined the possibility of human-to-human transmission, potentially accompanied by genomic alterations facilitating adaptation to the human host.

Among the 23 G4P[6] strains whose entire genome was determined, two distinct clusters (comprising two and three strains, respectively) exhibited identical or nearly identical genome sequences across all 11 segments. Two possibilities have been proposed to account for the detection of genetically identical porcine-derived RVA strains in different children: (i) multiple independent interspecies transmission events occurred from pigs harbouring the same strains or from environmental sources contaminated by them, or (ii) single spillover events were followed by subsequent, sequential human-to-human spreads. Due to the lack of documented contact between the affected children and pigs or pig-related materials, and no epidemiological links were found among the children themselves, it remains difficult to determine which scenario is more likely. However, even if human-to-human transmission did occur within these clusters, each strain was detected in only a few individuals over the 2-year surveillance period. This suggests that these strains did not establish sustained transmission chains and were likely eliminated from the human population.

In contrast, the remaining 18 G4P[6] strains exhibited distinct genome sequences, suggesting that each likely resulted from an independent interspecies transmission event from pigs or related environmental sources. Notably, none of the 23 G4P[6] strains analysed showed evidence of acquiring genome segments from human RVA strains. Taken together, these findings indicate that while limited human-to-human transmission may have occurred, the high detection rate of porcine-derived G4P[6] strains in Vietnamese children is more plausibly explained by frequent and repeated pig-to-human spillover events.

Supporting this interpretation, Papp *et al*. [1] reviewed the global distribution of G and P genotypes of RVA strains in livestock and reported that P[6] is the second most common P genotype in pigs worldwide, following P[7]. Although large-scale molecular epidemiological studies in Vietnam are lacking, available data from Hong Anh *et al*. [64] and Phan *et al*. [43] indicate that G4P[6] strains are predominant in pigs in southern Vietnam. This G4P[6] predominance, combined with environmental conditions in which humans and pigs live in close proximity, may facilitate the relatively high frequency of pig-to-human transmission events in Vietnam.

Another potential contributing factor is the molecular characteristics of the porcine RVA P[6], which may confer an advantage in infecting human hosts. The VP4 protein, particularly its VP8* domain, plays a key role in host range restriction by mediating glycan binding on the host cell surface. Phylogenetic analysis of P[6] sequences (Fig. 1b) revealed that porcine RVA-like P[6] strains form clusters distinct from those of typical human RVA-like P[6] strains, with evidence of geographic clustering in strain distribution. Based on this observation, we hypothesized that specific amino acid residues in the VP8* domain may underlie differences in glycan-binding specificity between porcine RVA-like P[6] and human RVA-like P[6], which in turn may explain geographic variation in the distribution of porcine-derived P[6] strains in human populations. Comparative analysis of the variable region of the VP8* domain between the 2 groups identified 15 amino acid residues that consistently differentiated porcine RVA-like P[6] from human RVA-like P[6] strains (Fig. 2, Table 6). Among these, five residues are known to be involved in glycan binding. In particular, residues 169–172 were highly conserved within each group (≥95% identity) but exhibited low or no similarity between groups, highlighting their potential role as group-specific molecular markers. Interestingly, in several Southeast and East Asian countries, most P[6] strains detected in humans harboured porcine RVA-like P[6] amino acids at positions 169–172 (HGGR), suggesting a porcine origin (Tables5  6). In contrast, in regions such as Africa and South Asia, where P[6] strains are more commonly found in human populations, P[6] strains predominantly exhibited the human RVA-like P[6] amino acid motif (FYNS) at these positions, consistent with their classification as typical human-derived P[6] strains.

The VP8* domain of the VP4 protein interacts with various host glycans, including sialic acids, gangliosides, histo-blood group antigens (HBGAs) and mucin core structures [61]. While some animal RVA strains depend on terminal sialic acids, most human and many animal RVA strains, including the human G4P[6] strain ST-3, are resistant to sialidase treatment [65], suggesting that asialoglycans such as HBGAs and mucin cores serve as primary receptors.

Major human RVA genotypes within the P[II] genogroup (P[4], P[6], P[8] and P[19]) interact with HBGAs and mucin cores in a genotype-specific manner [60]. HBGA expression is determined by glycosyltransferases encoded by the FUT2 (Secretor), FUT3 (Lewis) and ABO gene families and varies among populations, thereby influencing host susceptibility to infection. Secretor-positive individuals are susceptible to P[8] strains, whereas secretor-negative individuals are generally resistant [66,68]. In Burkina Faso, P[8] strains infected only Lewis- and secretor-positive children, whereas P[6] strains were predominantly detected in Lewis-negative children [69]. The relatively high prevalence of P[6] strains in African populations may be attributed to the high frequency (>30%) of the Lewis-negative phenotype, compared with frequencies below 10% in Europe and North America [70,73]. Although FUT3 genotype data are unavailable for Vietnamese individuals, a Lewis-negative frequency of 9.8% has been reported in Han Chinese in a neighbouring country [74], and most Vietnamese individuals are secretors (67.0% secretor-positive, 32.7% weak secretors and 0.3% non-secretors) [75]. Given the high prevalence of P[8] strains among Vietnamese children, the frequency of the Lewis-negative phenotype in the Vietnamese population may be relatively low.

HBGA expression is also developmentally regulated. Neonates are typically Lewis-negative during the first 1–2 months of life, with unbranched type 1 precursor glycans predominating in the gut [76]. Structural studies have suggested that the neonatal tropism of certain P[6] strains may reflect their high affinity for these immature HBGA precursors [77,78]. Consistently, P[6] strains are frequently detected in neonates in South Korea [79], and in one hospital-based study, all five P[6] strains were isolated from neonates, most of whom were Lewis b (Le^b^)-negative [80], supporting this structure-based hypothesis.

Structural studies show that VP8* proteins of the P[II] genogroup share a conserved glycan-binding region, the *βα* site, and interact with type 1 HBGAs and/or mucin core 2 [78,81,84]. VP8* of P[8] and P[4] also recognize Le^b^-containing HBGAs via a distinct ββ site [78,85]. While Le^b^ binding through the *ββ* site is well established for P[8] and P[4], evidence in P[6] and P[19] is limited and suggests only weak binding in some strains [81,85, 86].

Although glycan binding at the *βα* site is broadly conserved across the P[II] genogroup [77,78, 82, 87], genotype- and strain-level differences in binding affinity have been reported. Such genotype- and strain-specific binding patterns are particularly evident for the H type I precursor glycan, Lacto-N-tetraose (LNT) and for mucin core 2. P[8] and P[4] strains generally do not bind LNT, whereas some human- or porcine-origin P[6] and P[19] strains do [81,84, 85]. Notably, the human RVA P[6] strain BM11596 exhibits higher affinity for LNT than the porcine RVA-like human RVA P[19] strain NIV929893 [85]. In contrast, mucin core 2 binding is conserved in P[8], P[4] and P[19] genotypes [82,87] but is more variable among P[6] strains. Human RVA P[6] strains ST-3 and 5311142 lack detectable mucin core 2 binding [81,84], whereas the porcine RVA P[6] strain z84 and the porcine-like human RVA P[6] strain LL3354 retain this capacity [84].

Structural analysis of BM11596 complexed with LNT identified multiple *βα* site residues (W81, M167, F169, Y170, N171, S172, W174, F176, A183, T184, T185, Y187, S189, R209 and E212) involved in glycan binding [85]. Many are conserved across P[II] and mediate interactions with type 1 HBGAs and mucin core 2 [61,85]. As observed in this study, residues 167 and 169–172 differ consistently between human RVA P[6] strains (M167 and FYNS) and porcine or porcine-like human RVA strains of P[6], P[19], P[8] and P[4] (L167 and H/YGGR). These differences may contribute to variation in glycan-binding specificity between P[6] strains of different origins, as suggested by Sun *et al*. [84].

Based on these findings, we propose that human-adapted (human RVA-like) P[6] strains arose from porcine RVA P[6] ancestors and acquired substitutions in the VP8* *βα* site that increased affinity for H type I HBGAs and/or their precursors in humans, particularly Lewis-negative individuals, possibly accompanied by loss of mucin core 2 binding. In contrast, the Vietnamese P[6] strains analysed here retain porcine RVA-like residues, with no clear evidence of human-specific adaptation. Contemporary porcine RVA P[6] strains may exhibit lower affinity for H type I HBGAs while preserving mucin core 2 binding, a property that could facilitate spillover into humans; however, functional studies are required to test this hypothesis.

As discussed thus far, in Vietnam, porcine G4P[6] strains appeared to have partially overcome the initial steps of the species barrier. This raises the question of whether these strains may further evolve in the near future to acquire the capacity for sustained human-to-human transmission.

Both G9 and G12 genotypes are believed to have originated from porcine RVA strains [88]. Following the initial identification of a G9 strain (WI61, G9P[8]) in a child in the United States in 1983 [89], only sporadic human infections with G9 strains were reported for several years [90]. However, since the mid-1990s, novel G9 variants, primarily in combination with the P[8] genotype, have emerged and disseminated globally, establishing themselves as major human RVA strains [5]. Similarly, approximately a decade after the first detection of G12 strains in children in the Philippines in 1987 [91], G12 strains, typically associated with either P[6] or P[8], began to spread worldwide [92,95]. Currently, most G9 and G12 strains circulating among humans possess either the Wa-like or DS-1-like genotype constellation in their internal capsid and nonstructural genes [7].

More recently, during the past decade, bovine-like G8 and equine-like G3 strains have emerged and caused outbreaks in various geographic regions, becoming dominant in certain countries [54,96, 97]. These strains share the P[8] VP4 genotype and exhibit the DS-1-like genotype constellation (I2-R2-C2-M2-A2-N2-T2-E2-H2) for their internal capsid and nonstructural genes. These patterns suggest that efficient human-to-human transmission likely requires a VP4 genotype commonly found in human RVA strains, namely, P[8], P[4] or human RVA-like P[6], in combination with either a Wa-like or DS-1-like backbone gene.

If a porcine-derived G4P[6] strain were to cause a human epidemic in the future, it would likely arise as a reassortant, in which multiple genome segments from the original porcine RVA strain have been replaced with those from human RVA strains. In Vietnam, rotavirus infections in children peak during the dry season from November to April, although cases occur year-round. In contrast, porcine-derived RVA strains such as G4P[6] strains have been detected sporadically in humans, without clear seasonality (Fig. S3). The continuous circulation of human RVA strains throughout the year increases the likelihood of co-infections with porcine RVA strains, thereby facilitating reassortment and enhancing the potential for adaptation to human hosts via the acquisition of human RVA genomic segments. Moreover, if human-to-human transmission of porcine-derived strains is occurring, even at a limited scale as this study suggests, it could contribute to their adaptive evolution by enabling prolonged persistence within the human population.

Taken together with historical examples, reassortment events involving the acquisition of human RVA genome segments appear to be critical for animal-derived strains to adapt to human hosts. Therefore, surveillance should not overlook RVA strains with G genotypes commonly found in porcine RVA (e.g. G3, G4, G5 and G9), even if they carry P genotypes typical of human RVA strains, as such strains may represent early signs of host adaptation.

## Supplementary material

10.1099/mgen.0.001685 Uncited Supplementary Material 1.

10.1099/mgen.0.001685 Uncited Supplementary Material 2.

## Abbreviations

BIC: Bayesian information criterion
HBGA: histo-blood group antigen
LNT: Lacto-N-tetraose
ML: maximum likelihood
NJ: neighbour-joining
NSP: non-structural protein
RT-PCR: reverse transcription PCR
RVA: *Rotavirus alphagastroenteritidis*
ViPR: Virus Pathogen Resource
VP: viral protein
WHO: World Health Organization
