# Supplementary material for: Factors contributing to the frequent interspecies transmission of G4P[6] Rotavirus alphagastroenteritidis strains from pigs to humans in Vietnam: molecular epidemiological insights
Source: Microb Genom. 2026 Apr 24;12(4):001685. doi: 10.1099/mgen.0.001685 (PMC13108923; doi:10.1099/mgen.0.001685)
Supplement: Uncited Supplementary Material 1. [file mgen-12-01685-s001.pdf]

Fig. S1

A: VP7(G3, G5, G9)

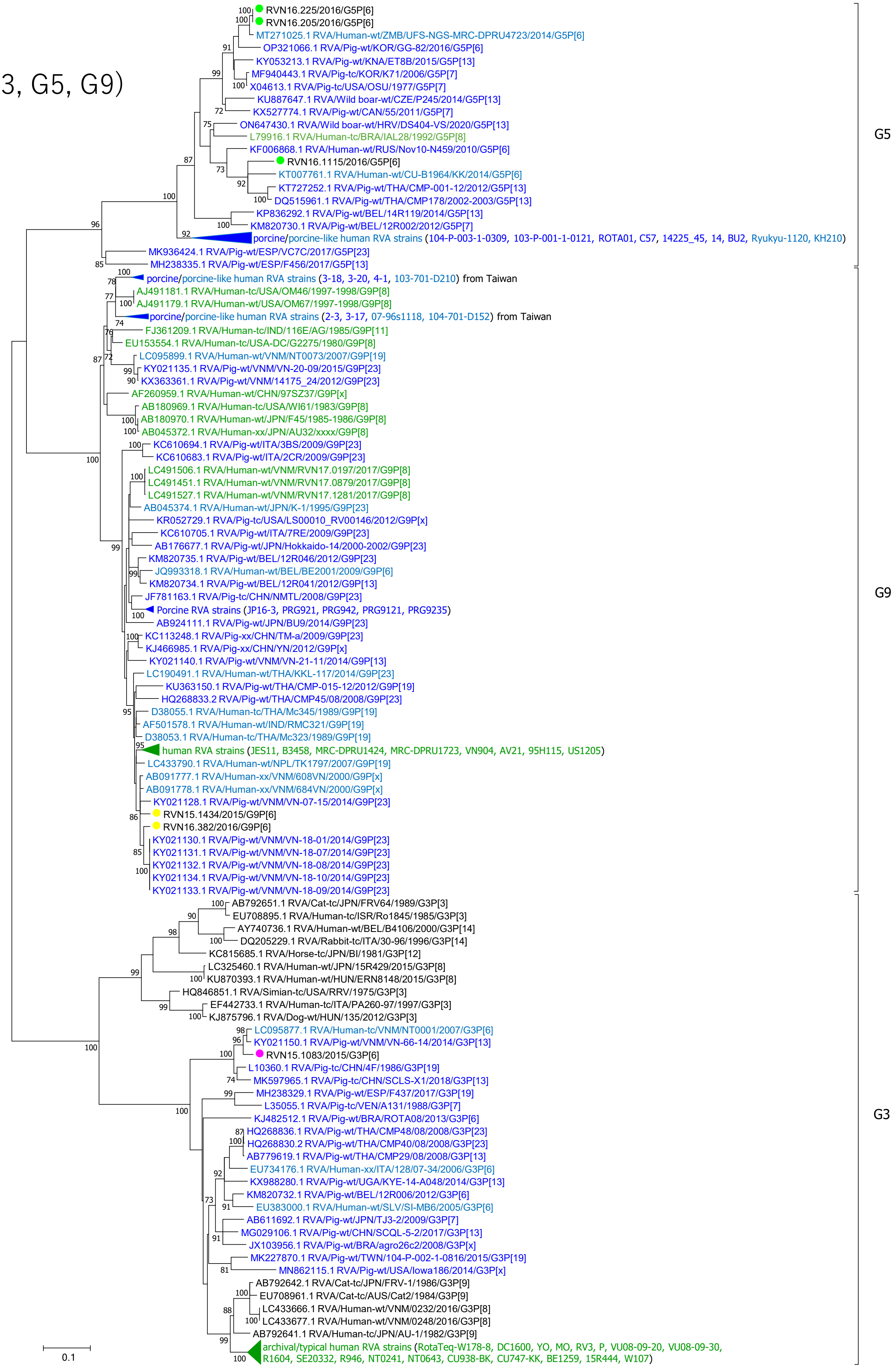

Fig. S1

B: VP6(I1)

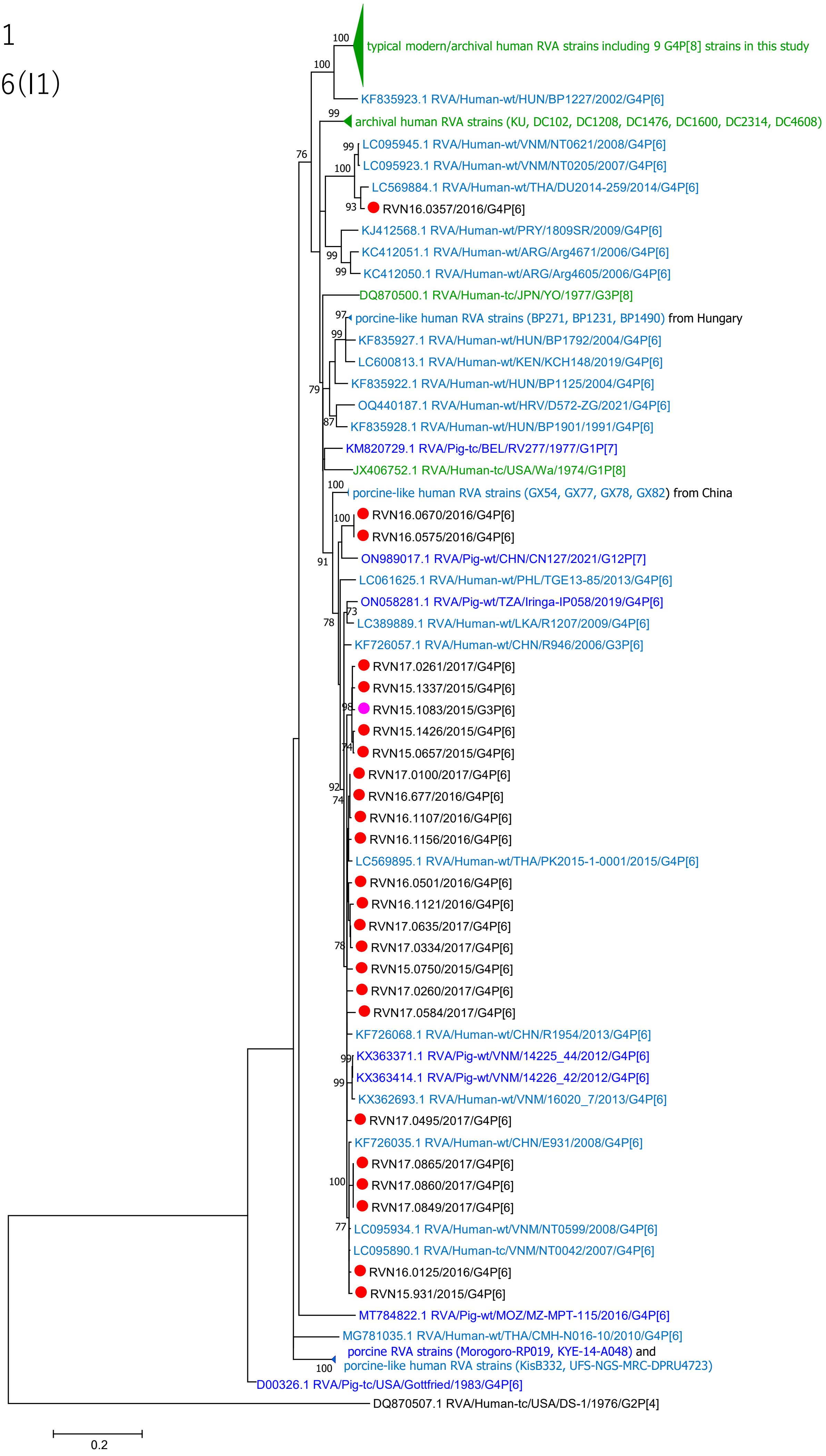

Fig. S1

C: VP1(R1)

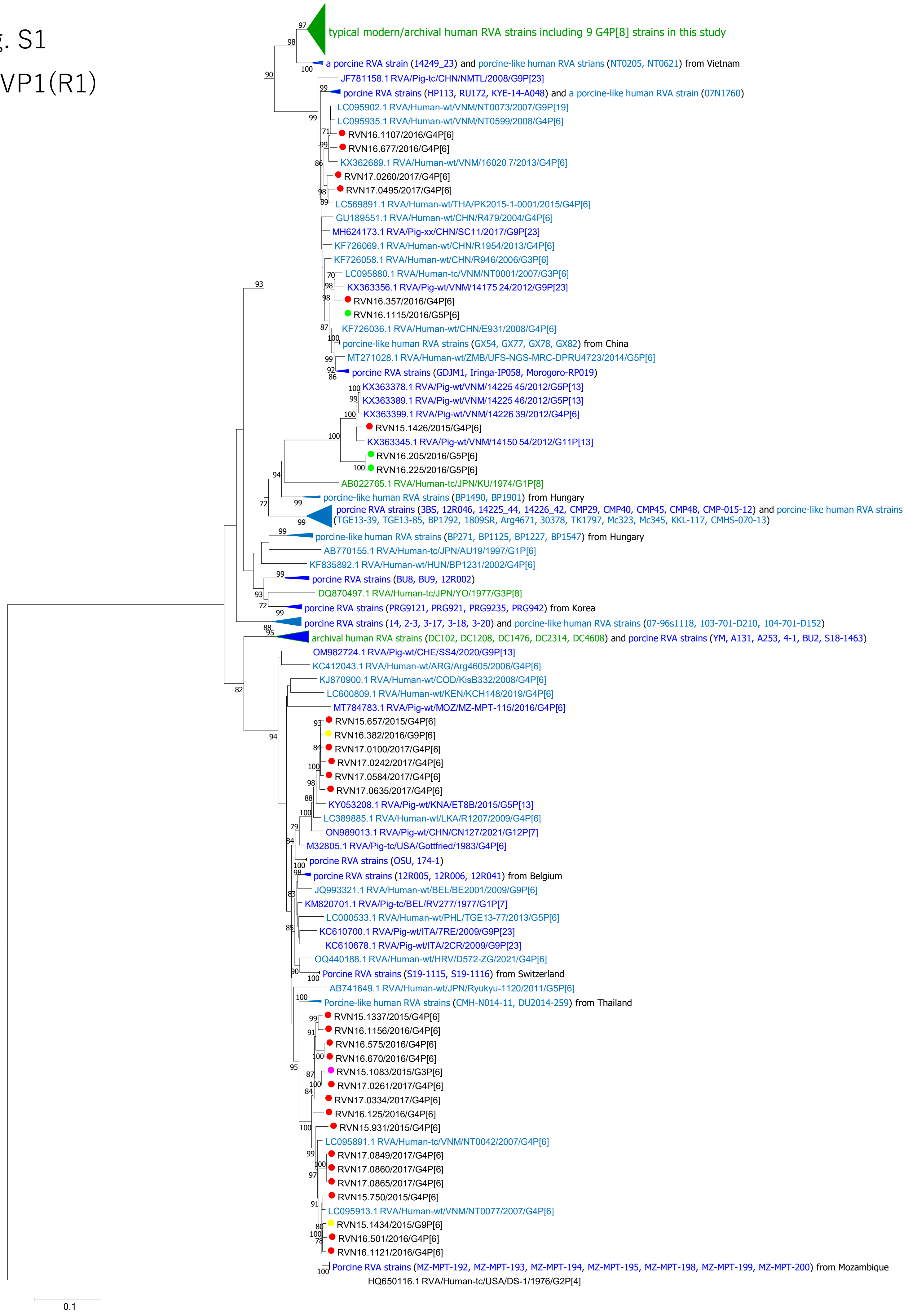

Fig. S1

D: VP2(C1)

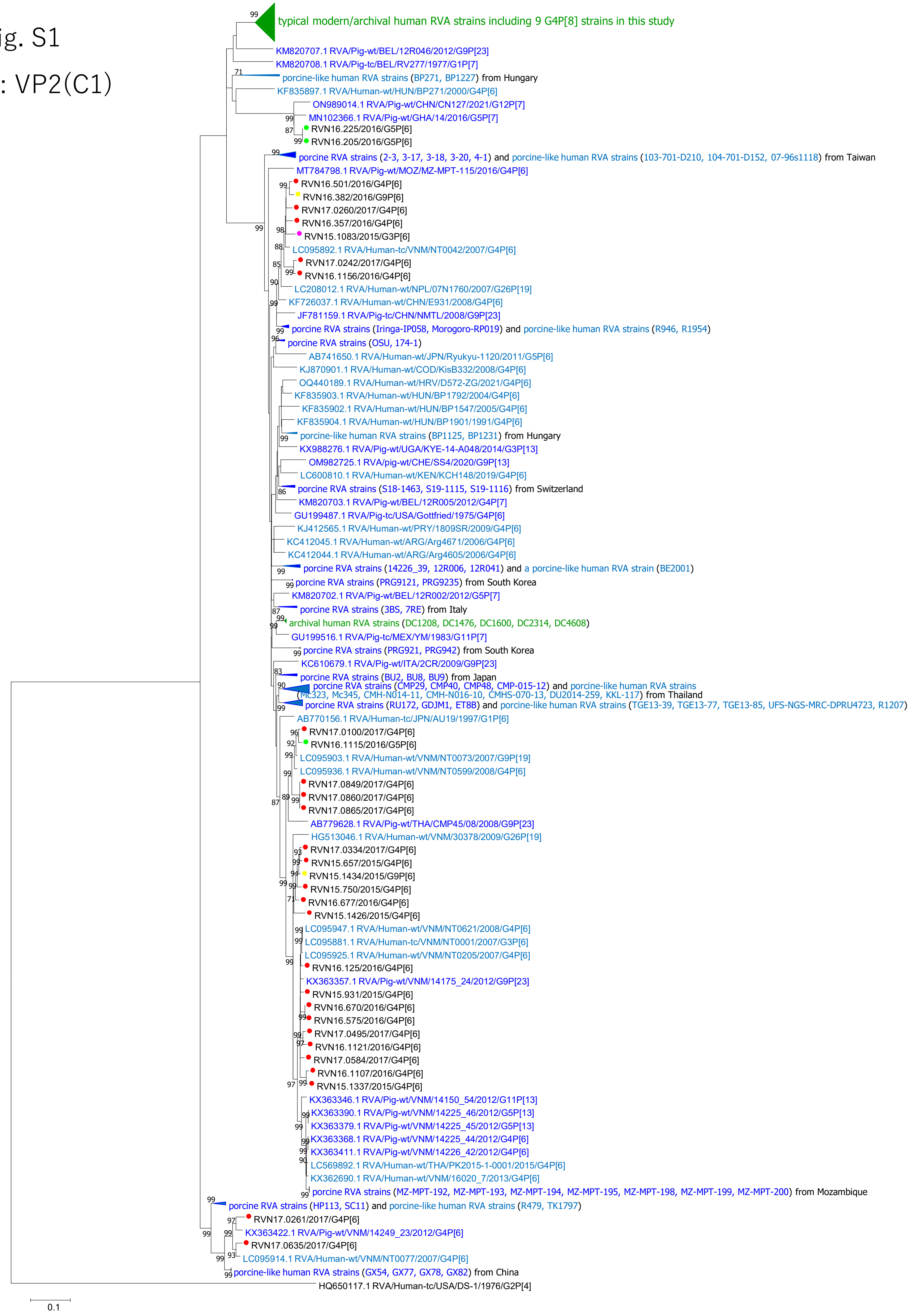

Fig. S1

E: VP3(M1)

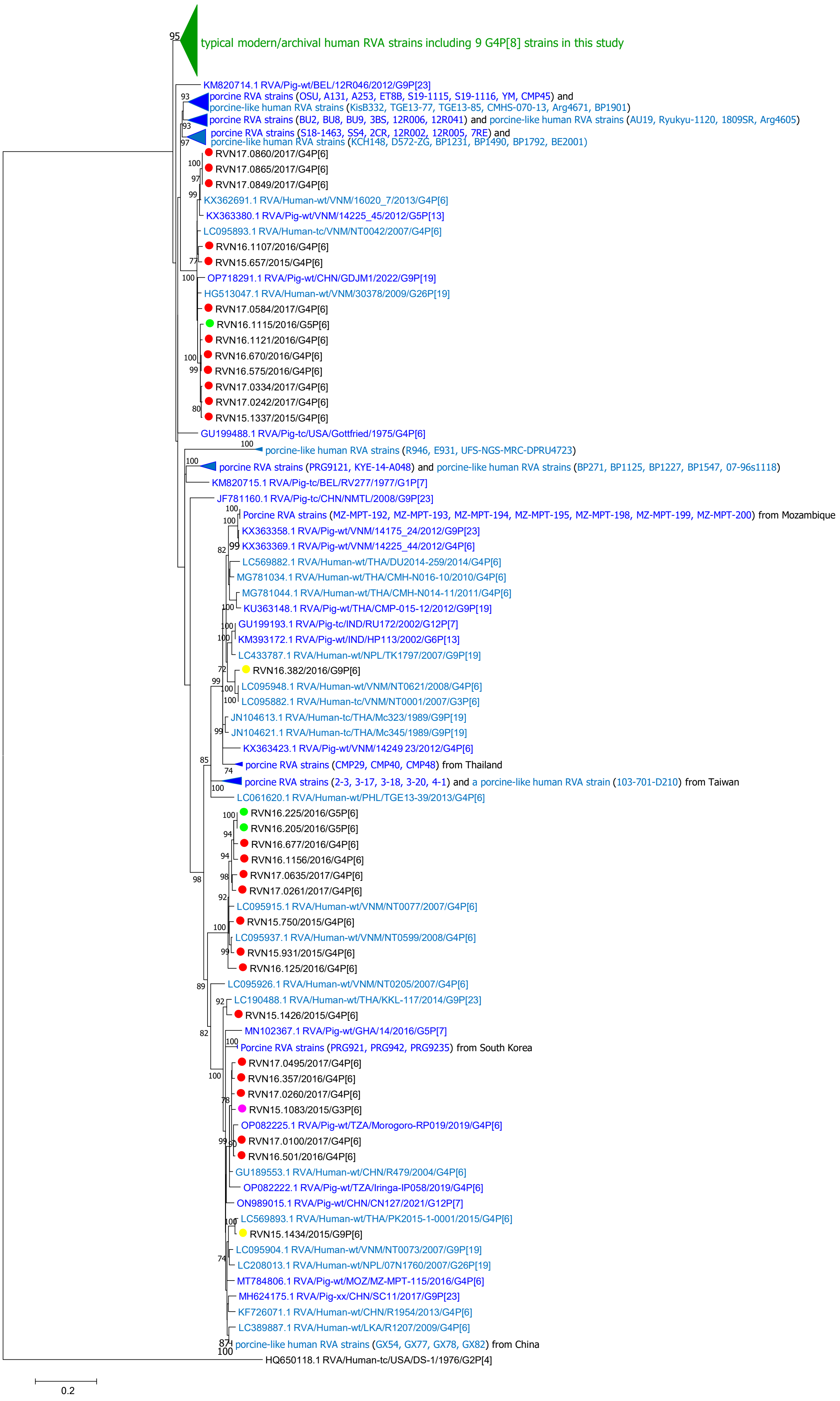

Fig. S1

F: NSP1(A1)

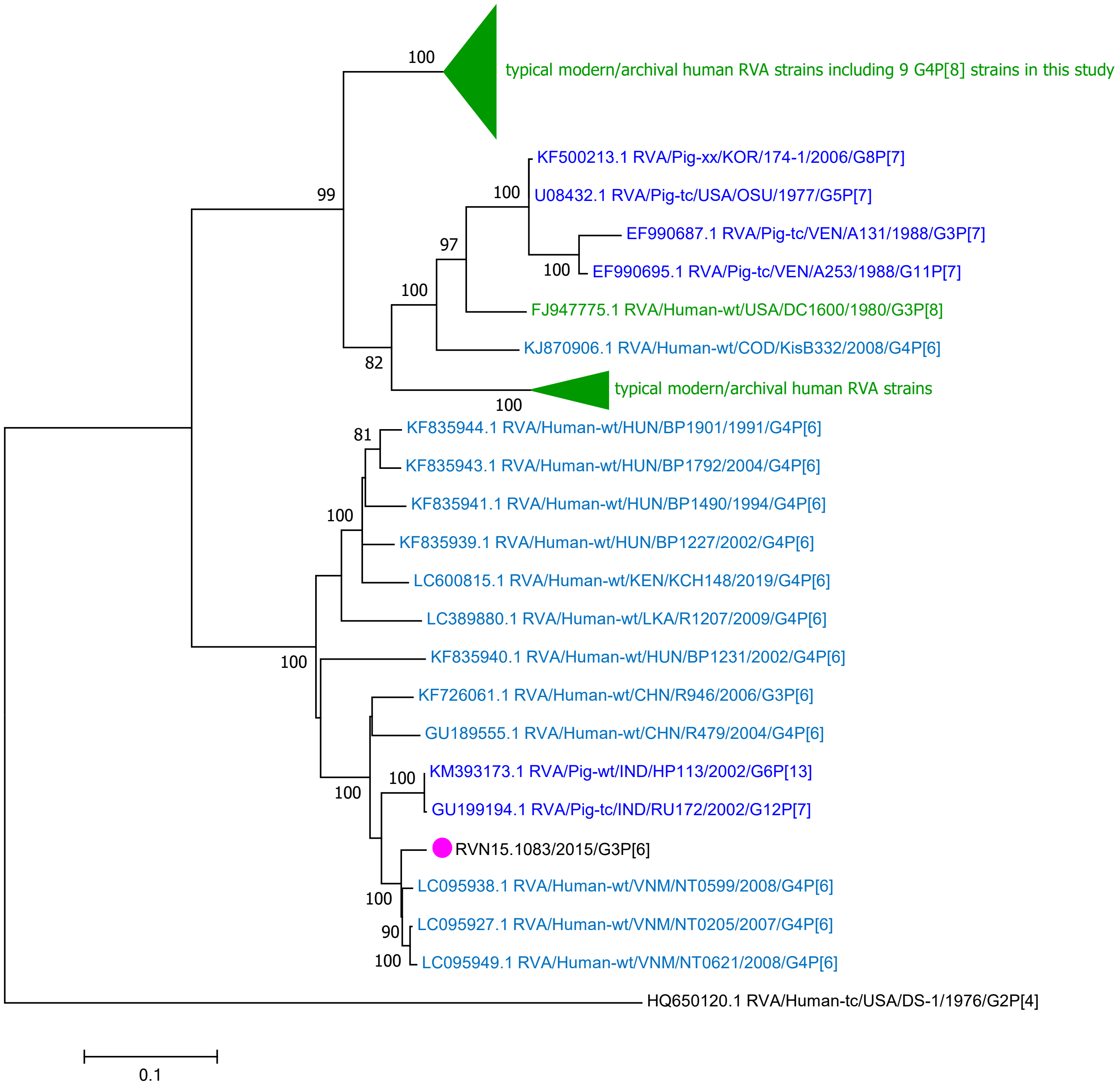

Fig. S1

G: NSP2(N1)

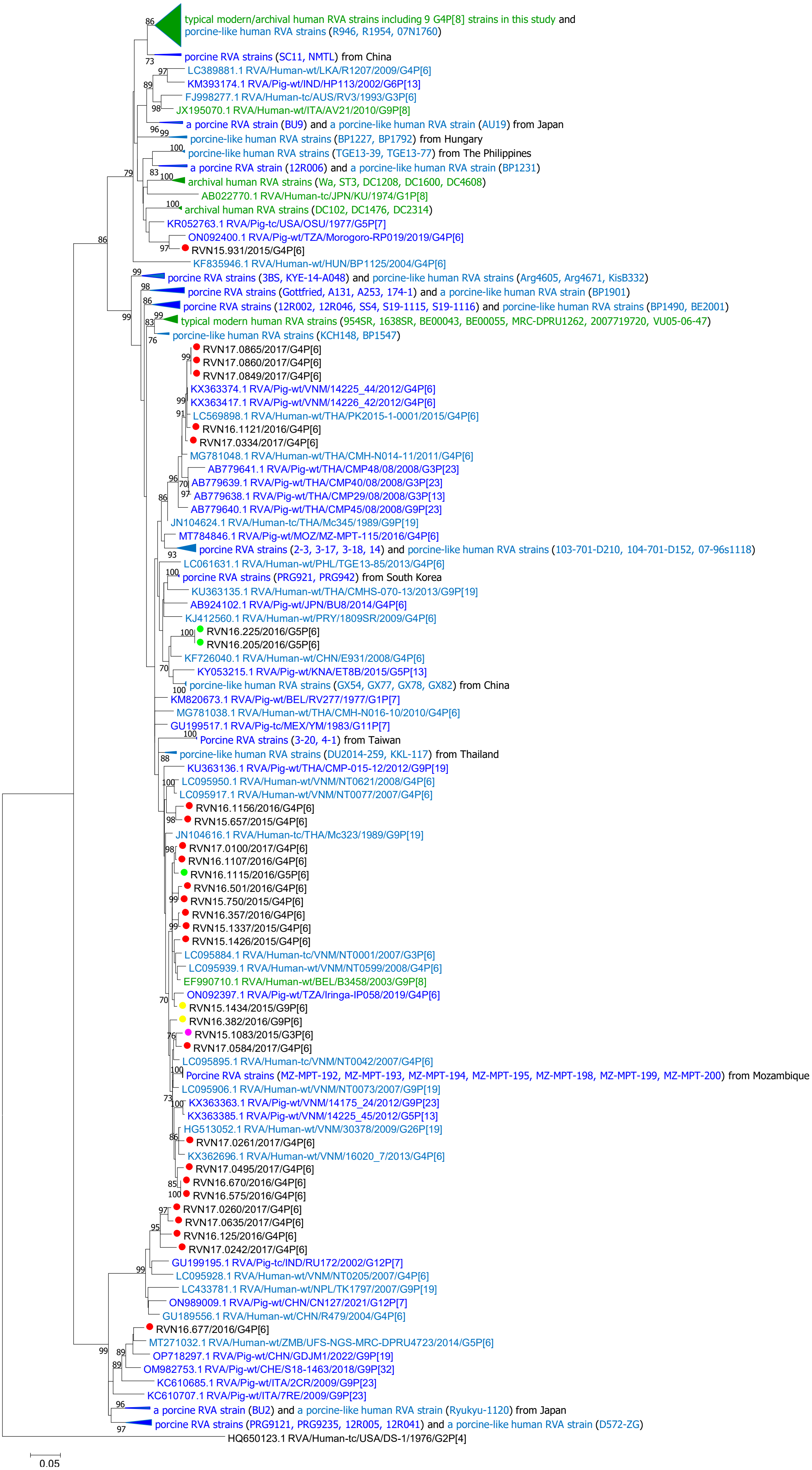

Fig. S1

H: NSP3(T1)

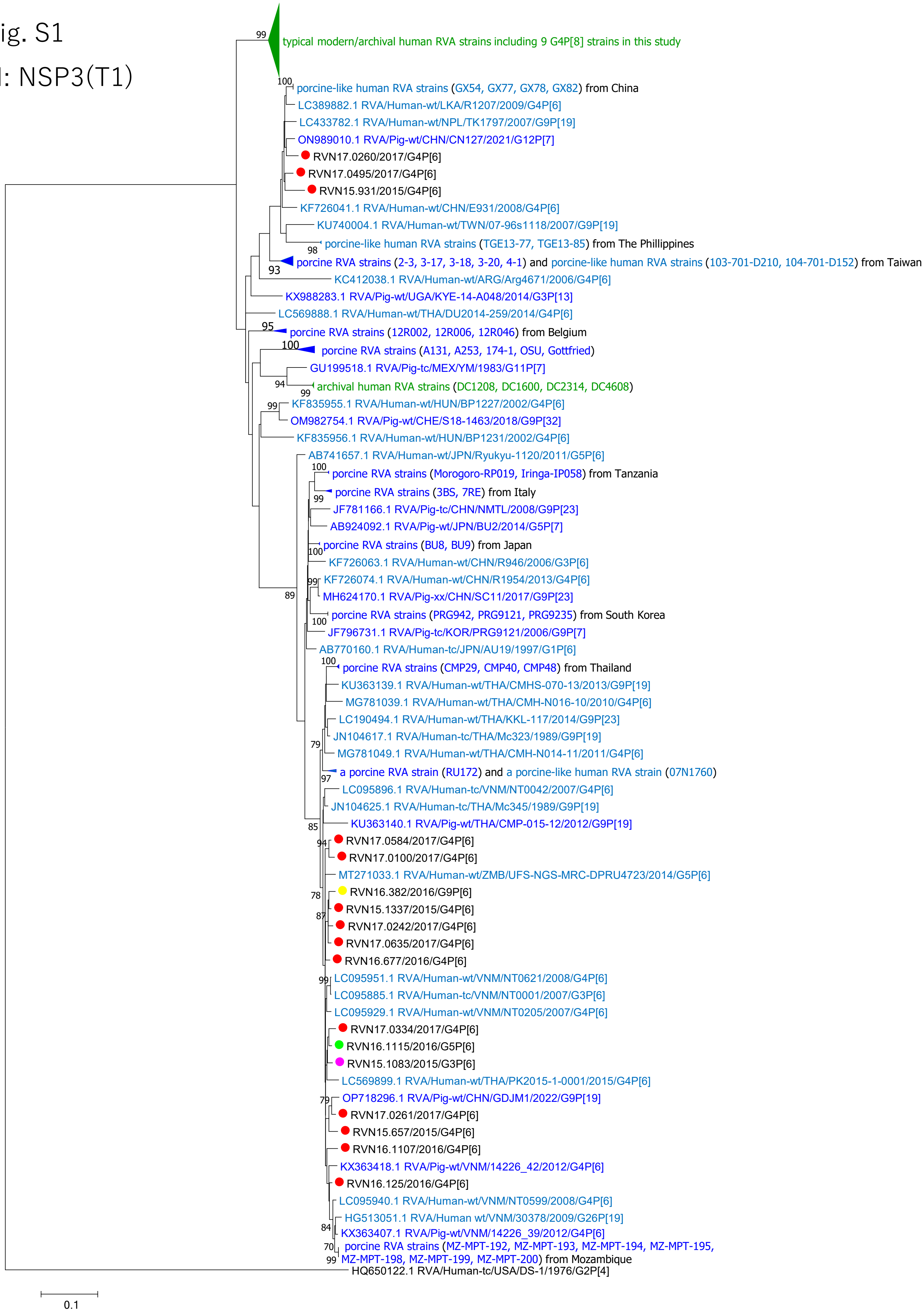

Fig. S1

I: NSP4(E1)

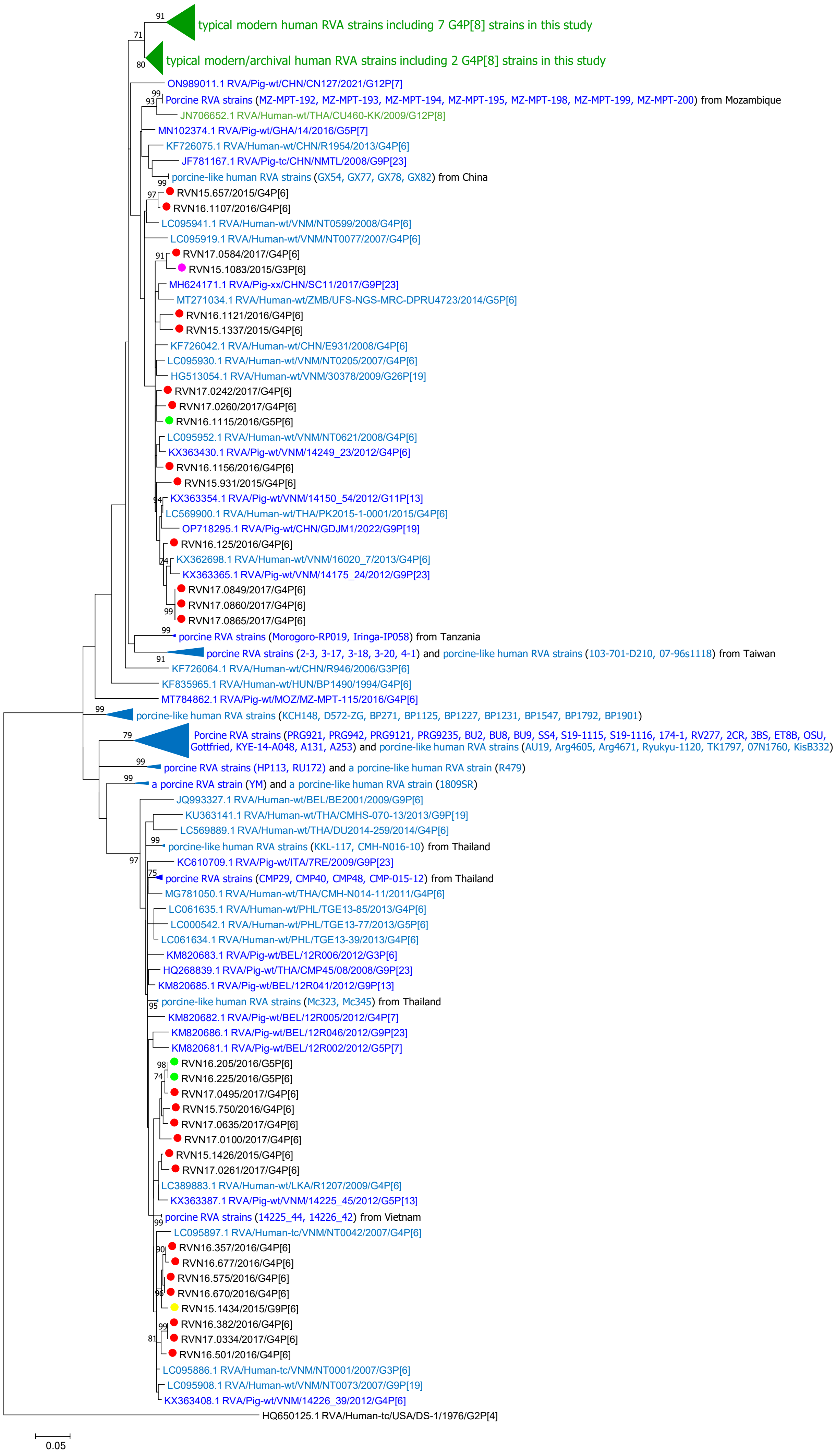

Fig. S1

J: NSP5(H1)

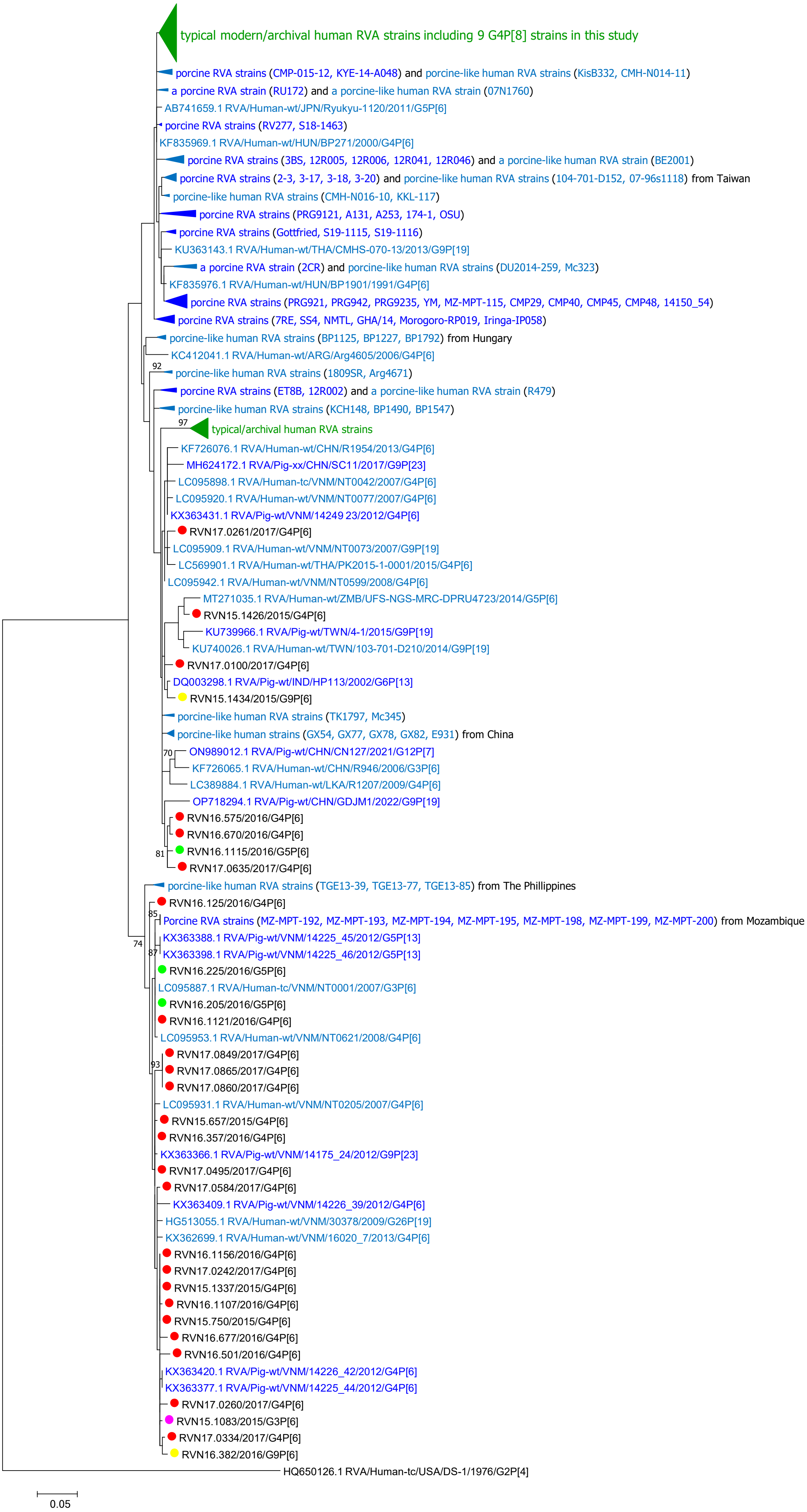

Fig. S1

K: VP6(I5)

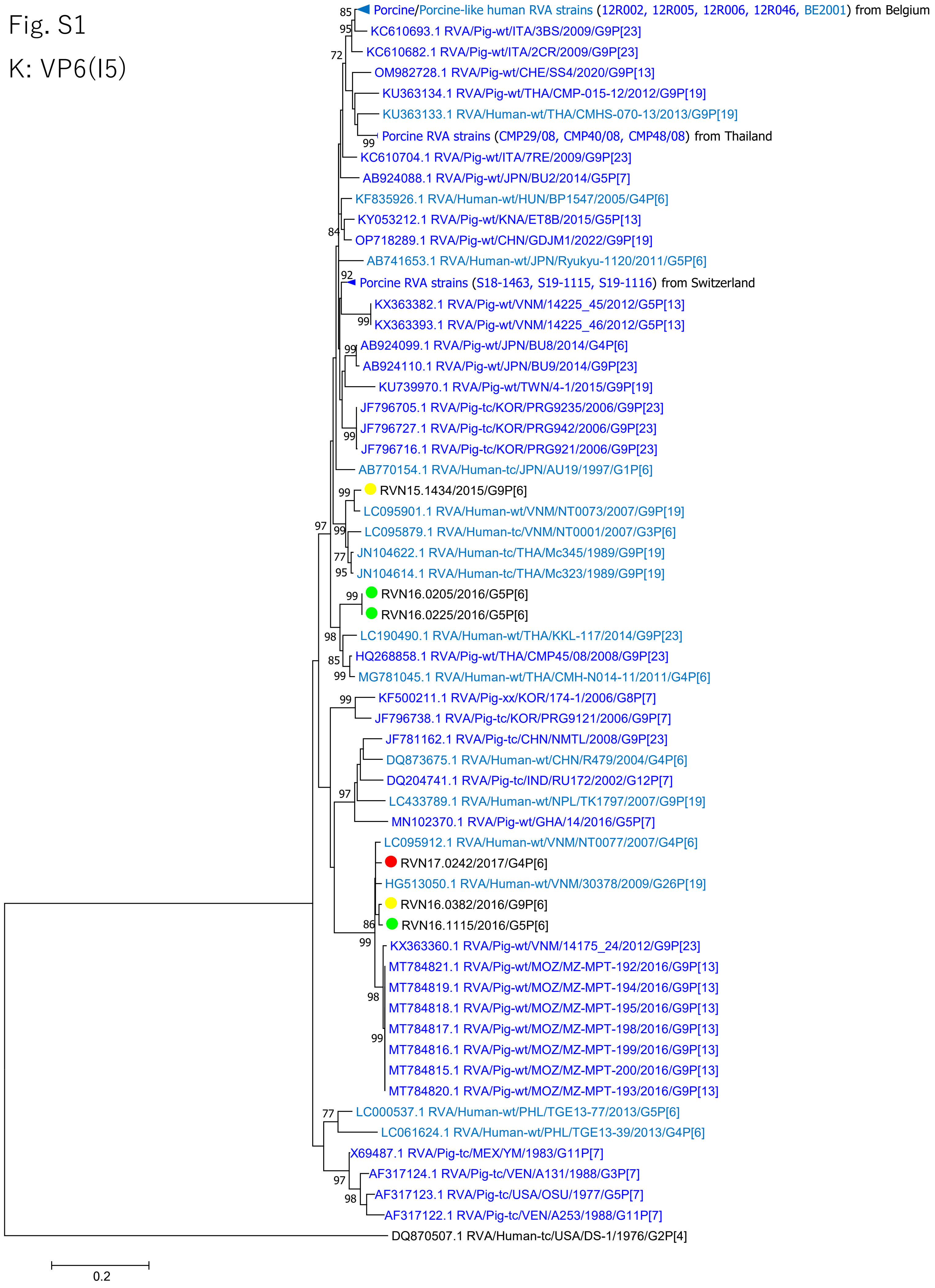

Fig. S1

L: NSP1(A8)

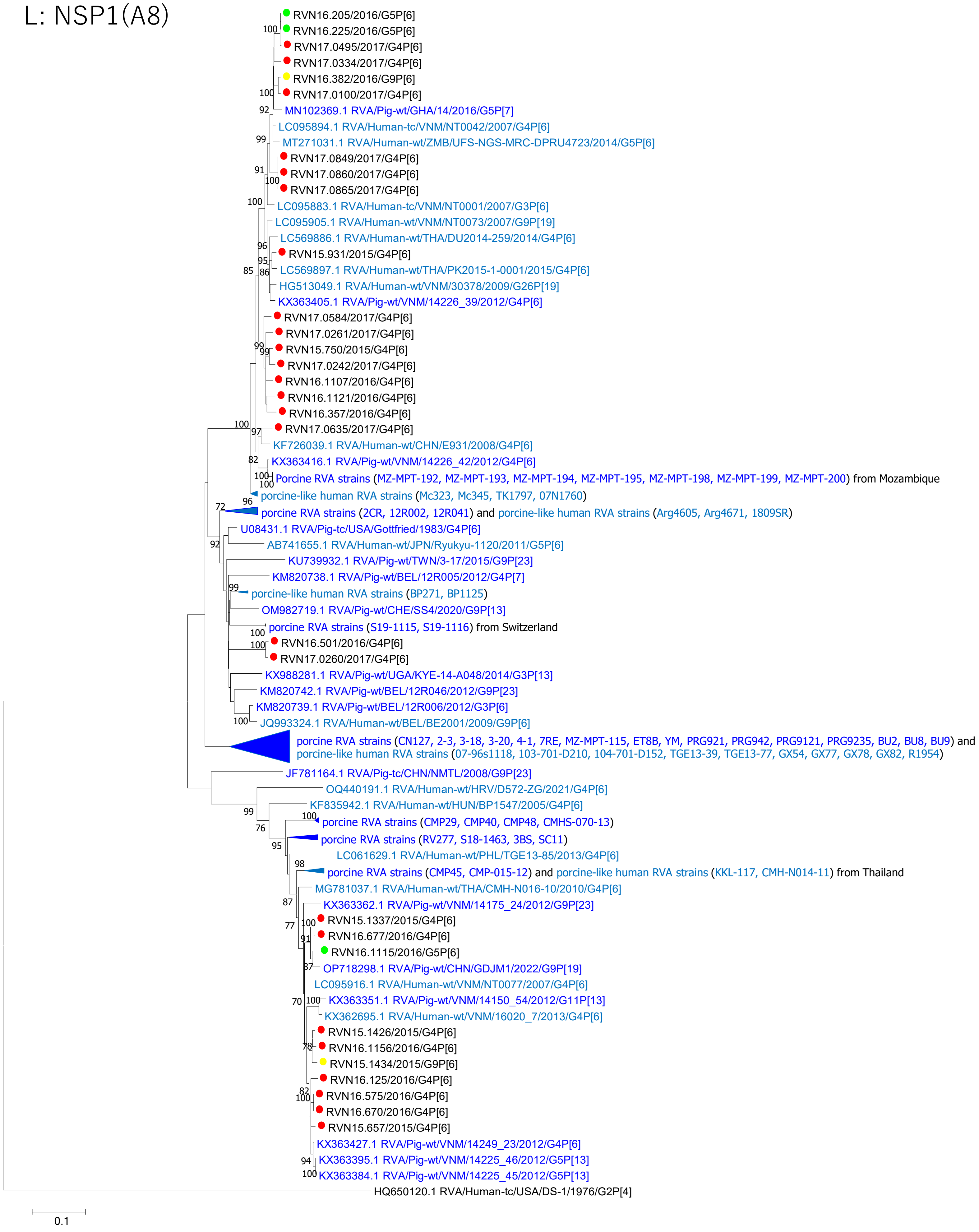

Fig. S1

M: NSP3(T7)

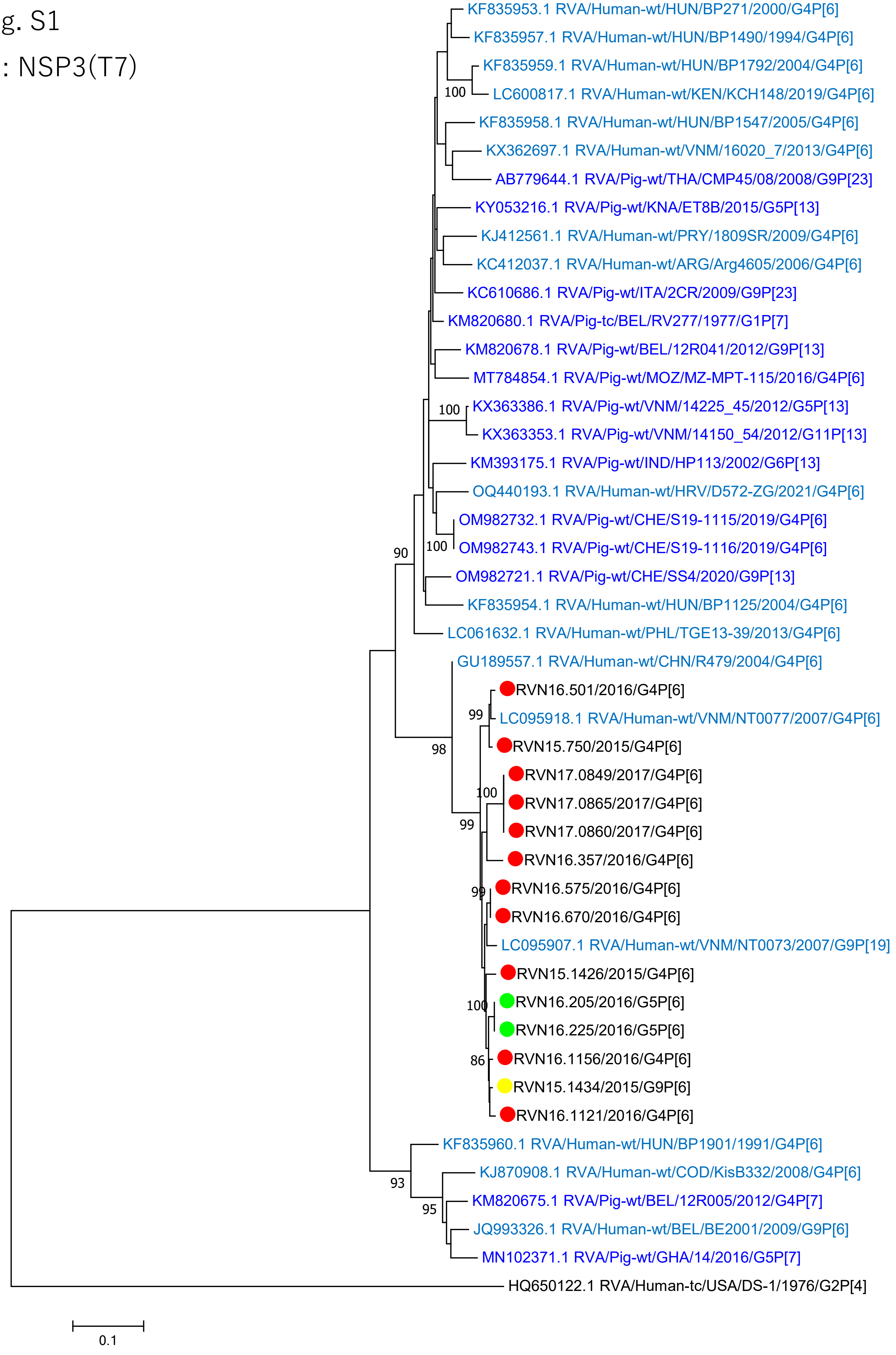

Fig. S1. Maximum likelihood (ML) phylogenetic trees of the G3/G5/G9 VP7 (A), I1 VP6 (B), R1 VP1 (C), C1 VP2 (D), M1 VP3 (E), A1 NSP1 (F), N1 NSP2 (G), T1 NSP3 (H), E1 NSP4 (I), H1 NSP5 (J), I5 VP6 (K), A8 NSP1 (L), and T7 NSP3 (M) genes from Vietnamese G4 and/or P[6] RVA strains and reference RVA strains. Phylogenetic trees were constructed using the ML method with 1,000 bootstrap replicates in MEGA7. Bootstrap values ≥70% are shown at the corresponding nodes. Scale bars indicate genetic distances, expressed as the number of nucleotide substitutions per site. Strain names of Vietnamese isolates sequenced in this study are shown in black, with colored filled circles indicating their G and P genotypes. Reference RVA strains are color-coded based on the host species in which they were detected and their presumed origin: human RVA strains from humans (green), porcine RVA strains from pigs (blue), and porcine-like RVA strains detected in humans (light blue).

Fig. S2

VP8*
(amino acids 65-223 of P[6])

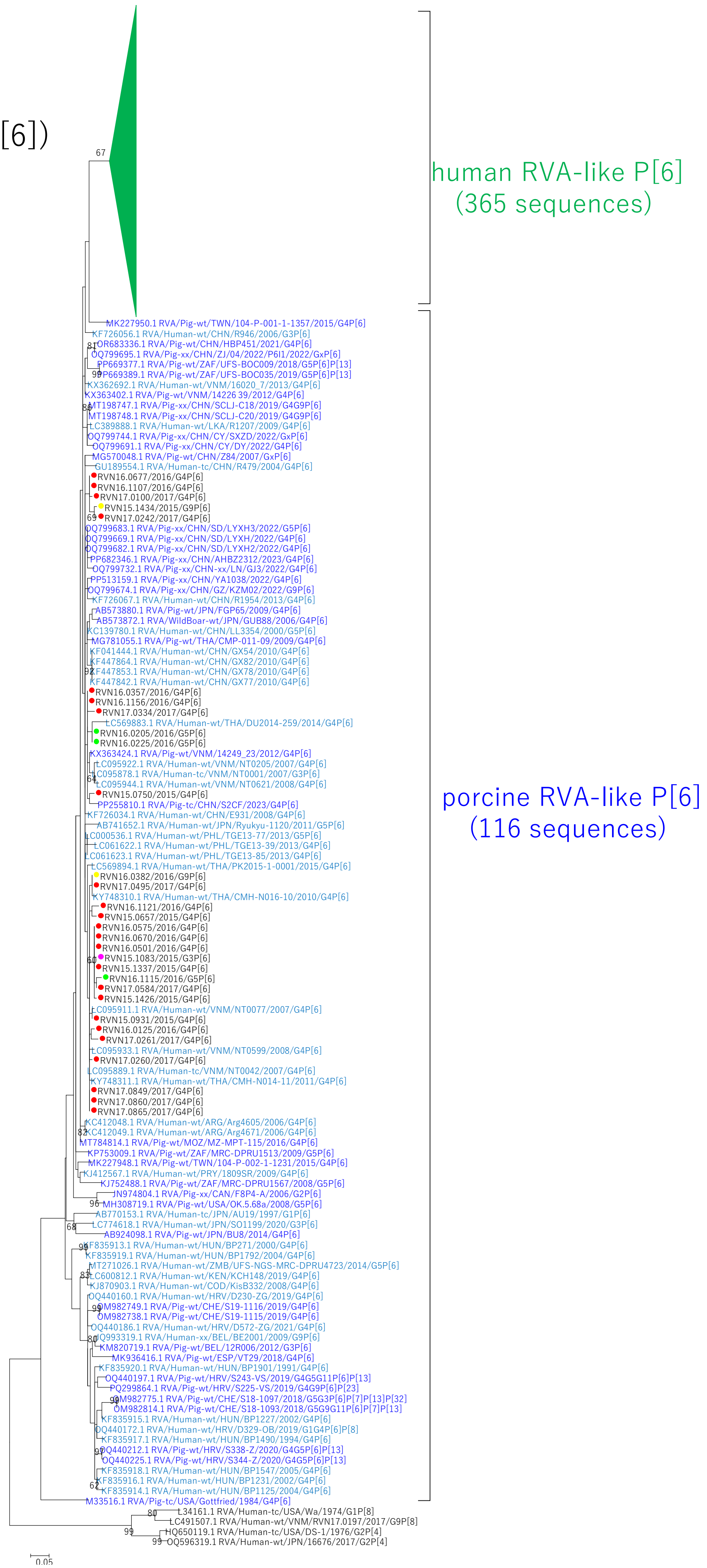

Fig. S2. A Maximum likelihood (ML) phylogenetic tree of 481 partial P[6] amino acid sequences (corresponding to the variable region of the VP8* protein, amino acids 65–223). The phylogenetic tree was constructed using the ML method with 1,000 bootstrap replicates in MEGA7. Bootstrap values ≥60% are shown at the corresponding nodes. Scale bars indicate the number of amino acid substitutions per site. Strain names of Vietnamese isolates sequenced in this study are shown in black, with colored filled circles indicating their G and P genotypes. Reference RVA strains are color-coded based on the host species in which they were detected and their presumed origin: human RVA strains from humans (green), porcine RVA strains from pigs (blue), and porcine-like RVA strains detected in humans (light blue). Based on tree topology, the P[6] sequences were classified into two groups: human RVA-like P[6] (n = 365) and porcine RVA-like P[6] (n = 116).

Fig. S3

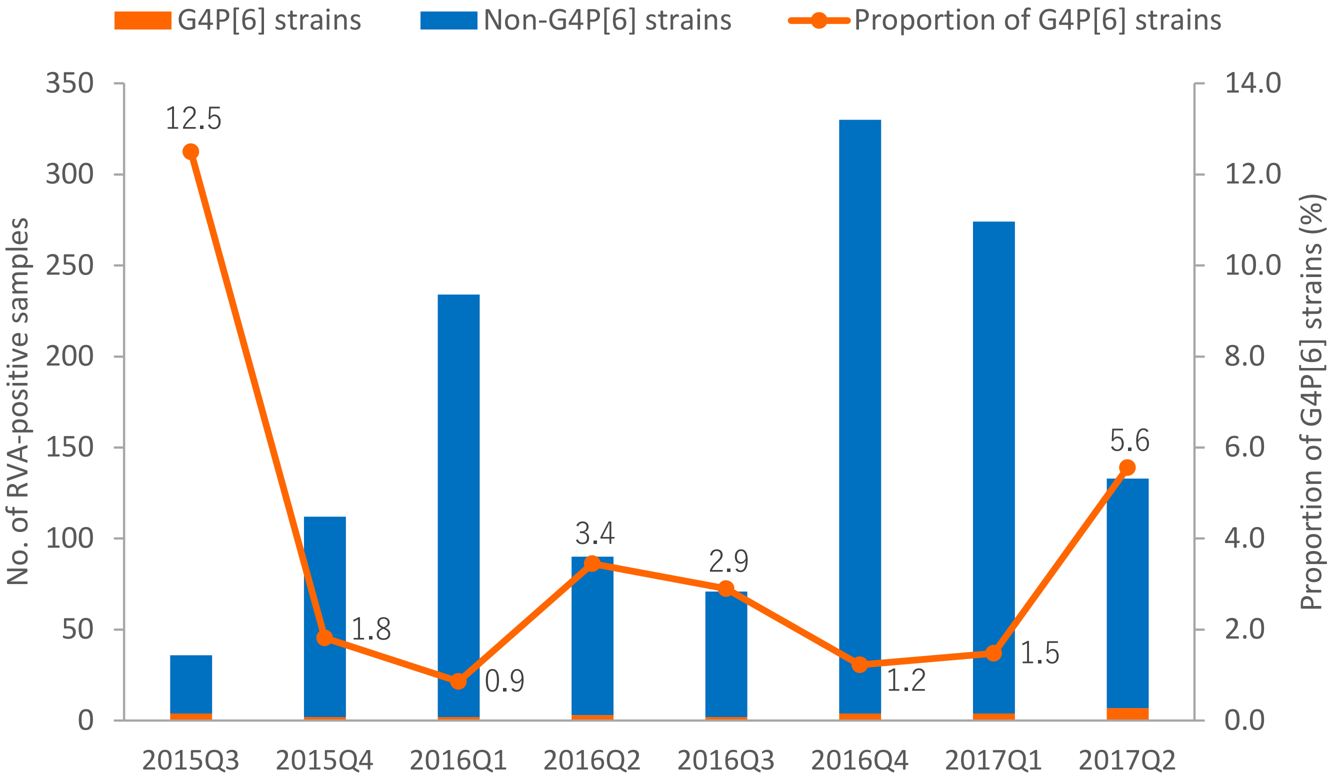

	2015Q3	2015Q4	2016Q1	2016Q2	2016Q3	2016Q4	2017Q1	2017Q2	Total
No. of G4P[6] strains	4	2	2	3	2	4	4	7	28
No. of RVA-positive samples	32	110	232	87	69	326	270	126	1252

Fig. S3. Number of rotavirus-positive samples and detection of G4P[6] strains by quarter, from July 2015 to June 2017. The bar graph shows the number of RVA-positive samples, with the blue portion indicating the number of samples containing only G4P[6] strains. The line graph represents the proportion of G4P[6] strains among the RVA-positive samples.
